# Supplementary material for: Establishing a core outcome set for treatment of uncomplicated appendicitis in children: study protocol for an international Delphi survey
Source: BMJ Open. 2019 May 22;9(5):e028861. doi: 10.1136/bmjopen-2018-028861 (PMC6538019; doi:10.1136/bmjopen-2018-028861)
Supplement: Supplementary data [file bmjopen-2018-028861supp001.pdf]

Online supplement 1.

S1. Completed COS-STAD checklist for PA-COS project

Domain	Methodology	Notes	Addressed on page number
Scope specification	1. The research or practice setting(s) in which the COS is to be applied	e.g., for application in research studies or for use in routine care	4.
	2. The health condition(s) covered by the COS	e.g., treatment of rheumatoid arthritis or screening for cancer	4.
	3. The population(s) covered by the COS	e.g., patients with advanced disease or children	4.
	4. The intervention(s) covered by the COS	e.g., all interventions, drug therapy, or surgical interventions	4.
Stakeholders involved	5. Those who will use the COS in research	e.g., clinical trialists or industry	6.
	6. Healthcare professionals with experience of patients with the condition	e.g., clinical experts, practitioners, and investigators with particular experience in the condition	6.
	7. Patients with the condition or their representatives	involve those who have experienced or who are affected by the condition (e.g., patients, family members, and carers).	6.
Consensus process	8. The initial list of outcomes considered both healthcare professionals' and patients' views.	consider the views of healthcare professionals and patients (most likely identified from literature reviews or interviews) when generating an initial list of outcomes for inclusion in the consensus process.	5. (No patient involvement)
	9. A scoring process and consensus definition were described a priori.	Although different consensus methods may be employed in different studies, to avoid any potential biases, COS developers should describe their consensus method a priori.	7, 8, 9.
	10. Criteria for including/dropping/adding	prespecify criteria for including, dropping, or adding new outcomes to avoid potential biases.	7, 8, 9.
	11. Care was taken to avoid ambiguity of language used in the list of outcomes.	consider the language used when describing outcomes in front of different stakeholder groups. An example of 1 approach taken is to include both lay and medical terms, with these previously piloted with the stakeholders.	7,8.
