# Supplementary material for: Establishing a core outcome set for treatment of uncomplicated appendicitis in children: study protocol for an international Delphi survey
Source: BMJ Open. 2019 May 22;9(5):e028861. doi: 10.1136/bmjopen-2018-028861 (PMC6538019; doi:10.1136/bmjopen-2018-028861)
Supplement: Supplementary data [file bmjopen-2018-028861supp002.pdf]

Online supplement 2.

S2. Search strategy for systematic review PA-COS

Appendicitis

“Appendix"[Mesh] OR appendix[tiab] OR appendix[ot] OR "Appendicitis"[Mesh] OR "Appendectomy"[Mesh] OR appendicit*[tiab] OR appendicit*[ot] OR appendectom*[tiab] OR appendectom*[ot] OR appendicectom*[tiab] OR appendicectom*[ot]

Children

child*[tw] OR schoolchild*[tw] OR infan*[tw] OR adolescen*[tw] OR pediatri*[tw] OR paediatr*[tw] OR neonat*[tw] OR boy[tw] OR boys[tw] OR boyhood[tw] OR girl[tw] OR girls[tw] OR girlhood[tw] OR youth[tw] OR youths[tw] OR baby[tw] OR babies[tw] OR toddler*[tw] OR teen[tw] OR teens[tw] OR teenager*[tw] OR newborn*[tw] OR postneonat*[tw] OR postnat*[tw] OR perinat*[tw] OR puberty[tw] OR preschool*[tw] OR suckling*[tw] OR picu[tw] OR nicu[tw]

3.2.2 Study selection

Selection of studies will be performed by 2 independent reviewers (MK, JF) according to the below mentioned in- and exclusion criteria. In case of disagreement between two reviewers, a third independent reviewer (RG) will make the final decision.

Inclusion/Exclusion Criteria:

All RCTs and systematic reviews/meta-analyses reporting the outcome of treatment of acute uncomplicated appendicitis will be included in this systematic review. By including systematic reviews that also report on non-comparative studies we expect to identify all reported treatment outcomes, including those from the relatively new field of non-operative management of uncomplicated appendicitis. Publications before January 2014 will be excluded. Only studies in children (<18 years of age) will be included. Studies only reporting on the outcome of treatment in complex or complicated appendicitis (gangrenous appendicitis, appendiceal mass, appendiceal abscess) will be excluded.

3.2.3 Data extraction

The two reviewers will extract the data independently using the predefined data extraction form shown in Appendix A. In case of disagreement a third reviewer will make the final decision. A risk of bias assessment of the individual studies is not applicable as we will not be using individual study data but only the reported outcomes. As diversity in terminology will be anticipated, we decided to initially report all outcome measure as mentioned in the original study. Outcome measures will be mapped independently by two reviewers and in case of disagreement a third reviewer will make the final decision. After data extraction of all studies is completed, a meeting of the study management group will be held between to discuss potential similarity between the outcome measures in order to assign an appropriate term for them.
