# Supplementary material for: Establishing a core outcome set for treatment of uncomplicated appendicitis in children: study protocol for an international Delphi survey
Source: BMJ Open. 2019 May 22;9(5):e028861. doi: 10.1136/bmjopen-2018-028861 (PMC6538019; doi:10.1136/bmjopen-2018-028861)
Supplement: Author's manuscript [file bmjopen-2018-028861.draft_revisions.pdf]

BMJ Open is committed to open peer review. As part of this commitment we make the peer review history of every article we publish publicly available.

When an article is published we post the peer reviewers' comments and the authors' responses online. We also post the versions of the paper that were used during peer review. These are the versions that the peer review comments apply to.

The versions of the paper that follow are the versions that were submitted during the peer review process. They are not the versions of record or the final published versions. They should not be cited or distributed as the published version of this manuscript.

BMJ Open is an open access journal and the full, final, typeset and author-corrected version of record of the manuscript is available on our site with no access controls, subscription charges or pay-per-view fees (http://bmjopen.bmj.com).

If you have any questions on BMJ Open's open peer review process please email editorial.bmjopen@bmj.com

BMJ Open

# Establishing a core outcome set for treatment of uncomplicated appendicitis in children: study protocol for a systematic review and international Delphi survey

| | |
|---|---|
| Journal: | *BMJ Open* |
| Manuscript ID | bmjopen-2018-028861 |
| Article Type: | Protocol |
| Date Submitted by the Author: | 28-Dec-2018 |
| Complete List of Authors: | Knaapen, Max; Emma Children's Hospital, Amsterdam UMC, University of Amsterdam, Vrije Universiteit, Paediatric Surgical Centre<br>Hall, Nigel; University of Southampton Faculty of Medicine, University Surgery Unit<br>van der Lee, Johanna H.; Emma Children's Hospital, Amsterdam UMC, University of Amsterdam, Paediatric clinical Research Office<br>Butcher, Nancy; Sick Kids, Child Health Evaluative Sciences<br>Offringa, Martin; The Hospital for Sick Children, University of Toronto, Department of Paediatrics<br>Van Heurn, Ernst; Emma Children's Hospital, Amsterdam UMC, University of Amsterdam, Vrije Universiteit, Paediatric Surgical Centre<br>Bakx, Roel; Emma Children's Hospital, Amsterdam UMC, University of Amsterdam, Vrije Universiteit, Paediatric Surgical Centre<br>Gorter, Ramon; Emma Children's Hospital, Amsterdam UMC, University of Amsterdam, Vrije Universiteit, Paediatric Surgical Centre<br>+ On behalf of the paediatric , appendicitis COS development group |
| Keywords: | delphi, appendicitis, children, core outcome set, paediatric, Global |
| | |

SCHOLARONE™
Manuscripts

# Establishing a core outcome set for treatment of uncomplicated appendicitis in children: study protocol for a systematic review and international Delphi survey.

Knaapen M, MD[1], Hall NJ, MD, PhD[2], Van der Lee JH, MD, PhD[3], Butcher NJ, PhD[4], Offringa M, MD, PhD[4, 5], van Heurn LWE, MD, PhD[1], Bakx R, MD, PhD[1], Gorter RR, MD, PhD[1] + On behalf of the paediatric appendicitis COS development group.

Minneci PC, MD, Svensson JF, MD, PhD, Luks FI, MD, PhD, St. Peter SD, MD, Abbo O, MD, Arnaud AP, MD, Adams S, MBBS, FRACS, Nah SA, MBBS, MRCS, Skarsgard ED, MD, Pierro A, MD, PhD, Emil S, MD, CM, Keijzer R, MD, PhD, Suominen JS, MD, PhD, Aziz DA, MD, FRCS

1. Emma Children's Hospital, Amsterdam UMC, University of Amsterdam, Vrije Universiteit, Paediatric Surgical Centre, Amsterdam, The Netherlands. Electronic address: rr.gorter@vumc.nl / r.bakx@amc.uva.nl / m.knaapen@uva.amc.nl / e.vanheurn@amc.uva.nl
2. University Surgery Unit, Faculty of Medicine, University of Southampton, Southampton General Hospital, Southampton, UK; Department of Paediatric Surgery and Urology, Southampton Children's Hospital, Southampton, UK. Electronic address: n.j.hall@soton.ac.uk
3. Emma Children's Hospital, Amsterdam UMC, University of Amsterdam, Paediatric clinical Research Office, Amsterdam, The Netherlands. Electronic address: j.h.vanderlee@amc.uva.nl
4. Child Health Evaluative Sciences, The Hospital for Sick Children Research Institute, Toronto, Canada; Electronic address: nancy.butcher@sickkids.ca
5. Department of Paediatrics, The Hospital for Sick Children, University of Toronto, Toronto, Canada. Electronic address: martin.offringa@sickkids.ca

Address correspondence to Max Knaapen, MD

| | |
|---|---|
| Address: | Emma Children's Hospital, Amsterdam UMC, University of Amsterdam, Vrije Universiteit, Paediatric Surgical Centre, Amsterdam. PO Box 22660, 1100DD Amsterdam, the Netherlands. |
| Email: | M.Knaapen@AMC.UvA.nl |
| Telephone: | +31-20-5665693 |


Word count: 4723

Funding statement: None
Competing interests statement: None to declare



For peer review only

# ABSTRACT

**Introduction**

Appendicitis is a global disease affecting roughly one in every 12 people in the world, with the highest incidence between ages 10 and 19 years. To date, a wide variety of health outcomes have been reported in randomized controlled trials (RCTs) and meta-analyses evaluating treatments for appendicitis. This is especially the case in studies comparing non-operative treatment to operative treatment. A set of standard outcomes, to be reported in all future trials, is needed to allow for adequate comparison and interpretation of clinical trial results and to make data pooling possible. This protocol describes the development of such a global core outcome set (COS) to allow unified reporting of treatment interventions in children with acute uncomplicated appendicitis.

**Methods and analysis**

We use current international standard methodology for the development and reporting of this COS. Its development consists of three phases: (1) Update the most recent systematic review on outcomes reported in uncomplicated paediatric appendicitis research, to identify additional outcomes, (2) Three-step global Delphi study to identify a set of core outcomes for which there is consensus between parents and (paediatric) surgeons, and (3) Expert meeting to finalize the COS and its definitions. Children and young people will be involved through their parents during phase two and will be engaged directly using a customized face-to-face approach.

**Ethics and dissemination.**

The medical research ethics committee of the Academic Medical Centre Amsterdam has approved the study. Each participating country/research group will ascertain ethics board approval. Electronic informed consent will be obtained from all participants. Results will be presented in peer-reviewed academic journals and at (international) conferences.

**Registration details**

The COS development project was registered with the COMET initiative in February 2018 (http://www.comet-initiative.org/studies/details/1119).


## Article Summary

## Strengths and limitations of this study

1. Globally relevant set of outcomes to be assessed in prospective research
2. International steering committee including patient representation
3. Involvement of parents and their children in choosing what to measure
4. Direct face-to-face involvement of young people, however, only in selected countries
5. Limited face-to-face consensus



For peer review only - http://bmjopen.bmj.com/site/about/guidelines.xhtml

# INTRODUCTION

Appendicitis is a common gastro-intestinal disease affecting roughly one in every 12 people in the world, with the highest incidence between ages 10 and 19 years[1,2]. While the incidence varies from country to country, appendicitis is a global disease[3]. In the last decade, there have been several developments in the treatment of appendicitis in children, with the most recent being non-operative treatment (NOT) for acute uncomplicated appendicitis. Studies investigating the effectiveness of NOT in children show promising results[4–7]. However, the selected primary (and secondary) outcomes vary widely, as reflected in recent systematic reviews assessing the efficacy and safety of NOT, this may contribute to their contradictory conclusions[4–8]. In the systematic review by Georgiou et al.[4], the need for universal outcome selection and reporting in appendicitis studies is emphasized. In general, it is recognized that clinical trials in children often lack outcomes that are appropriately chosen for this particular population[9].

Inconsistent selection and reporting of outcomes limits the ability to adequately compare and interpret clinical trial results. Furthermore, it hampers data pooling and subsequent meta-analysis. It also increases the risk of selective outcome reporting, a form of publication bias. This in turn jeopardizes the validity of results from individual trials, which feeds into subsequent systematic reviews[10] and meta-analyses, which are by nature retrospective, and therefore liable to various risks of bias[11,12].

As demonstrated by Hall et al. in 2015, a wide variety of outcomes has been reported in randomized controlled trials (RCTs) and meta-analyses reporting on the treatment of appendicitis in children[13]. In the 63 included studies, a total of 115 different outcomes were reported[13]. Hall et al. proposed the development of a Core Outcome Set (COS), which is a standardized collection of outcomes that should be measured and reported in all future trials[14]. Recently a study protocol was published for developing such a COS in the United Kingdom[15]. To overcome any limitations of a COS focused on UK-specific surgical practice, the aim of this study is to develop an international COS to be used for trials assessing the treatment of acute uncomplicated appendicitis in children. The development of this protocol and the international COS is being performed in conjunction with the UK research group. Outcomes considered important by patients and families are essential to a meaningful and complete COS[16]. Parent and patient representation in the development of this protocol was provided through the Dutch patient and parent Foundation: “Children and Hospital”. Furthermore, parents and patients will play a central role in the consensus process as a stakeholder group.

## Scope



For peer review only - http://bmjopen.bmj.com/site/about/guidelines.xhtml

For peer review only

We aim to reach a global consensus amongst patients, parents, researchers and physicians on the minimal set of core outcomes that should be measured and reported in all future clinical trials investigating any type of treatment for acute uncomplicated appendicitis in children, including surgical treatment, non-operative treatment or other treatment aimed at curing appendicitis.

# METHODS

In the development of this protocol, we adhere to the COS-STAD (Core Outcome Set-STAndards for Development) recommendations[17] and the COMET (Core Outcome Measures in Effectiveness Trials) handbook[18]. The completed COS-STAD checklist can be found in online supplement S1. The final core outcome set will be reported in accordance with the COS-Standards for reporting (COS-STAR) statement[19]. Involvement of patients and the public will be described using the GRIPP2 reporting checklist[20] (Guidance for Reporting on Involvement of Patients and Public). This study was registered with the COMET initiative (registration number: 1119) on February 11, 2018[21].

## Study design

The paediatric appendicitis COS (PA-COS) development will consist of three phases: (1) An update of the 2015 systematic review on outcomes reported in uncomplicated paediatric appendicitis research[13], in order to identify any additional outcomes used in trials that were published since the previous systematic review; (2) A three-step Delphi study to identify a set of core outcomes from those selected in the literature review. Development of the Delphi is performed according to the checklist by Sinha et al.[22] on the design and reporting of Delphi studies concerning COS selection; and (3) An expert panel meeting to ratify the final COS, including physicians, researchers and children/parent representatives. Children and young people will be involved through their parents during phase two and will be engaged directly using a customized approach.

## Steering Committee

An international steering committee has been established and consists of the following; the authors, a parent/patient representative of the Dutch Foundation: “Children and Hospital”, and the lead local investigator of each participating centre (paediatric appendicitis COS development group). The steering committee will agree on the final version of the protocol at the start of the project and will provide input throughout the duration of the project. The steering committee members will also be involved in the development of the final COS. Within the steering committee, a smaller study management group has been appointed



For peer review only - http://bmjopen.bmj.com/site/about/guidelines.xhtml

which will convene during regular (videoconference) meetings.

## Systematic review: Treatment outcomes

Hall et al. performed a systematic review of RCTs and meta-analyses reporting treatment outcomes in children with appendicitis up to April 2014[13]. The 115 unique outcomes were collapsed into a total of 38 standardized outcome terms. We will update the systematic review to identify any additional reported outcomes in clinical trials or systematic reviews. All RCTs and systematic reviews/meta-analyses reporting treatment outcomes of acute uncomplicated appendicitis in children (<18 years of age) published between January 1$^{st}$ 2014 and November 23$^{th}$ 2017 will be included. The final review will follow the PRISMA reporting guideline[23]. We will search the Cochrane Central Register of Controlled Trials, MEDLINE and EMBASE with the help of a clinical librarian. Additional information on the search strategy/study selection and data extraction can be found in online supplement S2. Studies only reporting outcomes of treatment in complex or complicated appendicitis (for example - gangrenous or perforated appendicitis, appendiceal mass, appendiceal abscess) will be excluded.

After data extraction, a meeting of the study management group will be held to discuss potential similarity between the outcomes in order to assign an appropriate standardized outcome term for them. Outcome terms will be mapped to four core areas (death, life impact, resource use, pathophysiological manifestations) in accordance with the methods from the OMERACT FILTER 2.0[24]. Although Hall et al.[13] chose to list the adverse events as a separate core area, we will reclassify these outcome terms to one of the four core areas (Table 1.). We will however, label adverse events of treatment separately, as the OMERACT filter suggests[24]. A meeting of the study management group will be held to discuss potential similarity between outcomes and to assign an appropriate outcome term for similar outcomes. Outcomes that are only found once and are not generalizable can be excluded (e.g. the width of lateral thermal damage of the mesoappendix after appendectomy). Grouping the outcomes under a common outcome term aims to arrive at a manageable and cohesive list of outcomes that is appropriate as a basis for the Delphi questionnaire.



For peer review only - http://bmjopen.bmj.com/site/about/guidelines.xhtml

**Table 1. Outcome Core Areas**

| Core Area | Example(s) |
|---|---|
| Life impact | *Quality of life, loss of ability to work* |
| Resource use | *Length of hospital stay, healthcare costs, societal cost* |
| Pathophysiological Manifestations | *Biochemical parameters, organ function, (ir)reversible manifestations (complications, pathology results)* |
| Death | *Death* |

## Stakeholders and recruitment

### 1) Children and Young People

Children and young people (5-18 years) who have been treated for acute uncomplicated appendicitis in the preceding 24 months, either with initial NOT or with surgery. Children less than 5 years old are excluded since different outcomes might be appropriate in this very young age group. Also, uncomplicated appendicitis is much less common in young children than in older children. Furthermore, there are no studies in which children below the age of 5 are treated non-operatively. Children will be engaged (indirectly) through their parents in the Delphi questionnaire, and directly through a customized face-to-face approach in selected countries. For the invited children, considering the complexity of the subject and methodology, age is limited to 12-18 years.

### 2) Parents

Parents of children and young people (5-18 years) treated for acute uncomplicated appendicitis either with initial NOT or with surgery in the preceding 24 months or during the initial phase of the study. Parents will be asked to discuss the answers they provide with their child whilst filling out the Delphi questionnaire. Parents will be invited to participate by their child's treating physician or their designate in each participating country/hospital. Participants will be identified retrospectively by


For peer review only - http://bmjopen.bmj.com/site/about/guidelines.xhtml
For peer review only

contacting patients that were treated in the past 24 months or prospectively by inviting patients to participate directly after they have completed their treatment.

3) **(Paediatric) Surgeons**
General and/or paediatric surgeons who care for children in the specified age group will be asked to participate. Surgeons will be identified and invited by the local coordinators in each participating country. These local coordinators are research groups that have previously registered a clinical trial on uncomplicated appendicitis in children. This should allow for inclusion of physicians that also have experience in research on the treatment of appendicitis.

**Participating countries and research groups**

It was decided to invite research groups that are currently conducting clinical trials on the treatment of acute uncomplicated in children. Groups were identified through www.clinicaltrials.gov by searching (January 2017) for 'appendicitis' with an age limitation of 5-18 years. Studies with a mixed population (children and adults) were excluded. Studies that had been completed before 2014, had not been updated since 2015, or with incomplete registrations, were excluded. We found 111 trials, of which 12 trials assessed the treatment of uncomplicated appendicitis in children. Groups from the Netherlands, USA, Canada, Australia, Sweden, Finland, UK, France, Italy, Israel, Japan, Singapore and Malaysia were identified. Some trials included hospitals from multiple countries.

## Sample size

There is no rationale for determining the number of respondents to invite for a Delphi study[18]. A minimum of seven respondents per stakeholder group is suggested to have a large enough group to allow for a consensus process[25]. Taking into account that only some invited participants will register for the Delphi and that not all respondents will complete all rounds of the Delphi (attrition), a minimum of 40 respondents per stakeholder group per country will be invited. There will be no maximum. In case the number of respondents per country is significantly higher than other countries, we will consider a weightage per country in the analyses. We anticipate that this sample will be large enough to reflect all relevant opinions.

## Delphi study

International online Delphi study

The Delphi method is an effective tool for reaching consensus in a large group without the need for face-to-face contact[26]. The use of sequential questionnaires which are answered



For peer review only

anonymously by stakeholders is an established method for reaching consensus in a group of experts[22]. Questionnaires will be sent using DelphiManager[27], a web-based system designed for Delphi studies. The questionnaires will be open simultaneously to all respondents of the participating countries. After each round, the responses of all participants are shared anonymously in accordance with the Delphi principle.

The list of outcomes from the systematic review will be formatted into questions with extensive additional information per outcome. The Delphi questionnaire will originally be formulated in English and will be translated if required. Translation will only be performed by native speaking professionals.

Participants will be asked to score the importance of each outcome using a 1 to 9 Likert scale as recommended by the Grading of Recommendations Assessment, Development and Evaluation (GRADE) working group[28] and COMET initiative[18]. A score of 7-9 indicates a critical outcome for assessing the effect of a treatment, 4-6 important but not critical, 1-3 indicates an outcome with low importance for assessing the treatment effect. It will also be possible to select an “unable to score” option, which is especially of importance if parents do not feel equipped to score certain outcomes. The questionnaires will be piloted by a group of laypersons (n=10) to check for ambiguity and readability.

Delphi round one

Participants will be divided into two stakeholder groups: parents (with their children), and surgeons. Parents will be asked to discuss the answers they provide with their child whilst filling out the Delphi questionnaire. Baseline characteristics (age, country) will be ascertained. Parents will be asked if their child was treated with non-operative or operative treatment, time between registration and the first diagnosis of appendicitis and if their treatment was with or without complications. Surgeons will be asked their speciality (paediatric, general, abdominal, other), workplace (academic, teaching hospital, non-teaching hospital), experience with non-operative treatment, experience in research regarding appendicitis in children.

All participants will be asked to score all previously identified outcomes according to their perceived importance for assessing the treatment effect. In the first round there will be an option to suggest additional outcomes not yet listed.

Participants will have between four and eight weeks to complete each round, depending on the response rate. In that time they will receive two reminder emails as long as they have not yet replied to the questionnaire.

Delphi round one: analysis



For peer review only

Results will be analysed **by stakeholder group and for all participants** using descriptive statistics. Outcomes will be analysed separately for each stakeholder group, as there is evidence that patients are likely to assign importance to outcomes differently than surgeons[29], which has the potential to influence eventual outcome selection.

"Consensus-in" will be defined as:

- Greater than 70% of participants in **both** stakeholder groups scoring the outcome as 7-9 and less than 15% in **both** stakeholder groups scoring the outcome as 1-3.
- Greater than 90% of participants within **one** stakeholder group scoring the outcome as 7-9. This implies that these outcomes are highly regarded by an individual stakeholder group, and should also be included[18].

"Consensus-out" will be defined as:

- Greater than 70% of participants in **both** stakeholder groups scoring the outcomes as 1-3 and less than 15% of participants in **both** stakeholder groups scoring the outcome as 7–9. Consensus-out can only be reached when there is consensus across **both** stakeholders groups.

Outcomes that do not meet any of these criteria will be defined as "no consensus". A stratified analysis will be performed to check for skewing as a result of divergent opinions from a single country or surgeons with or without research experience.

At the end of round one, there will be a meeting of the study management group to assess whether an alteration in the Delphi study is appropriate. If additional outcomes are suggested by Delphi participants, each outcome will be assessed by the study management group to determine whether it is indeed new and to which category it should be classified. Wording of the Delphi questionnaire will be adjusted if misinterpretation is suspected.

Delphi rounds two and three

All participants who have completed the previous round will be asked to participate in the next round. Only outcomes that have not been defined as "consensus-in" or "consensus-out" during the previous round will be presented in the following rounds to all participants. Outcomes for which there was "consensus-in" only within a single stakeholder group, will still be presented to the other stakeholder group to evaluate whether consensus can be achieved in both stakeholder groups. An overview of included and excluded outcomes will be available. The outcomes for which there is no consensus and the newly suggested outcomes from the previous rounds will be presented with the participants' individual score and the median scores from each stakeholder group combined with a histogram showing the scoring



For peer review only - http://bmjopen.bmj.com/site/about/guidelines.xhtml

distribution. Participants will be asked again to score all remaining outcomes in the same manner as in round one.

Delphi rounds two and three analysis

Results will be analysed per stakeholder group and for all participants, using descriptive statistics with the same definitions for consensus in/out as in the first Delphi round, including a stratified analysis. After the second round, there will be a meeting of the study management group to assess the need for alteration in the Delphi study, and to decide whether or not to proceed with a third Delphi round, assuming consensus between **both** stakeholder groups on more than 80% of the outcomes, and more than five outcomes with consensus in. To give an estimate of the degree of agreement between respondents, the width of the interquartile range of the median ranking score will be calculated, potentially ranging from 0.00, meaning complete agreement, to 8.00, meaning least possible agreement. This will be calculated for both the individual stakeholder groups as well as the entire group of respondents after the final round.

## Face-to-face engagement of young people

We wish to check for discrepancies of opinion between parents answering the Delphi together with their child and children who are interviewed directly. For this, a form of in-person interaction will be organised with young people (12-18 years) who have been treated for appendicitis. They will be asked to comment on the preliminary COS selection established at the end of the Delphi study, and to suggest additional outcomes and comment on outcomes that did not make the preliminary COS selection. This will either be done by a short, face-to-face, one round questionnaire involving only outcomes relevant to children/young people, or in the form of a small consensus meeting (prioritization meeting) before finalizing the definitive COS. Due to feasibility, the face-to-face engagement will take place in selected countries and separate ethics board approval will be obtained as appropriate in those countries.

## Consensus discussion

If adequate consensus (we aim to achieve consensus on at least one outcome per OMERACT core area) is reached in the Delphi study, we will organise a face-to-face expert panel meeting with selected individuals with the purpose to ratify a pragmatic and well-defined set of outcomes. A secondary aim of this meeting is to enhance support and implementation of the final COS.

The meeting will be held at an international conference for paediatric surgery. Through purposive sampling, approximately 30 “experts” from across all stakeholder groups, including



For peer review only - http://bmjopen.bmj.com/site/about/guidelines.xhtml

physicians, researchers and children/parent representatives, will be invited to participate in a face-to-face meeting with the Steering Committee. Journal editors and healthcare commissioners will also be invited to attend in an observational capacity with the purpose of promoting implementation and to provide comments on the final list of outcomes.

In the event that adequate consensus cannot be reached in the Delphi process, we will organise a formal face-to-face consensus meeting or teleconference. In that case, we will select an appropriate representation of all stakeholder groups from the panel members that participated in the Delphi study.

## Final COS development

The goal is to achieve a pragmatic COS that is applicable and feasible for all future trials that evaluate the treatment of uncomplicated appendicitis in children. To achieve the goal of a pragmatic COS we aim to arrive at a maximum of 10 outcomes. As a minimum, we aim to have at least one outcome per core area. If consensus is reached on more than 10 outcomes, the 10 outcomes with the highest level of consensus will be considered part of the suggested COS. Highest level of consensus depends on whether there is consensus in both stakeholder groups, the median score that was appointed to the outcome, and the interquartile range of the median score as an estimate of the degree of consensus.

Only outcomes for which consensus is reached internationally will be selected. To test for country bias, stratified analyses of the Delphi results will be performed. The results from the face-to-face engagement of young people will be taken into account for the final COS selection and will be reported separately. If there is no consensus between patients, parents and healthcare professionals, an outcome can still be selected if there is clear consensus within a single stakeholder group. These will be reported separately. The final COS will be categorised according to the four core areas.

## Ethics and dissemination.

Ethics

The medical research ethics committee of the Academic Medical Centre Amsterdam confirmed that the Dutch Medical Research Involving Human Subjects Act (WMO) does not apply to this study and that complete approval of this study by the committee is not required. Each participating country/research group will be asked to obtain ethics board approval or confirm that ethics board approval it not required. Electronic informed consent will be obtained from all participants. The face-to-face engagement of young people (12-18 years) will take place in selected countries and separate ethics board approval will be obtained, as appropriate.



For peer review only - http://bmjopen.bmj.com/site/about/guidelines.xhtml

For peer review only

Data collection and confidentiality

All data will be handled confidentially and in accordance with the Dutch Personal Data Protection Act and the European General Data Protection Regulation (GDPR). DelphiManager[27] will be used for the online questionnaire. After informed consent from all participants only limited identifying information (name, email) will be ascertained during registration. This information will be stored separately from the answers given in the questionnaire and will only be used for the purpose of direct feedback and reminder emails. Access to personally identifiable data will be strictly limited.

Dissemination

Dissemination of the results will be accomplished by publication in an international peer-reviewed scientific journal and by presentations at (international) conferences. By inviting as many of the principal investigators who are currently involved in research on uncomplicated appendicitis in children, we aim to optimize implementation of the final COS. By involving journal editors and healthcare commissioners in the face-to-face consensus discussion, we aim to ultimately have the COS introduced as a requirement in future outcome reporting on the treatment of uncomplicated appendicitis in children.

# DISCUSSION

## Strengths and limitations of this study

### Outcomes selection

The selection of potential outcomes will be done systematically and will provide a selection for the first Delphi questionnaire that reflects most issues pertinent to the treatment of uncomplicated appendicitis. By including systematic reviews/meta-analyses that also report on non-comparative studies, we expect to identify all reported treatment outcomes, including those from the relatively new field of NOT for uncomplicated appendicitis.

To be able to arrive at a manageable list of outcomes that is appropriate for a Delphi study, the number of outcome terms needs to be somewhat limited. In order to achieve this, the outcomes derived from our systematic review will be merged in case of similarity. If outcomes are not generalizable and only reported once, they will be excluded. This will be proposed and prepared by two independent reviewers and discussed in the study management group. However, the merging of outcomes will inevitably lead to some loss of detail.

### Global consensus

In order to reflect the views of different stakeholders, a variety of groups will be part of the development of this COS. This is not only the case on a national level, but also on an international level, related to, for example, differences between countries in resources,



For peer review only - http://bmjopen.bmj.com/site/about/guidelines.xhtml

treatment practises for acute uncomplicated appendicitis, and cultural differences. By also involving patients and parents from the participating countries we hope to correct for these differences[30]. Involving members from different countries will not only lead to the development of a COS that reflects the opinions of the international community, it should also lead to an internationally applicable “minimal” COS. However, selecting the participating countries on the basis of their involvement in research on appendicitis in children is a limitation. This choice was made on the basis of feasibility. Researchers in the field of uncomplicated appendicitis have an interest in the development of a COS and have the network to help carry out the Delphi study. With our current selection we will, however, have participants from four different continents. Our means of selection has another advantage. Since non-operative treatment is an important research subject in childhood appendicitis, we aim to include surgeons and parents who have experience in that field. As non-operative treatment is still experimental in most of the world, we need surgeons and patients who have been involved in such research.

**Limited face-to-face consensus**

If consensus is reached in the Delphi study we will not be organising a formal consensus meeting. The Delphi method can be used for reaching consensus in a group of respondents without the need for face-to-face contact. There is a risk of bias if a face-to-face consensus meeting leads to selection of only participants who are able to attend the meeting, which is especially a problem in a global consensus procedure. There are also problems regarding language barriers in an international consensus meeting. To check for interpretation errors in the Delphi method and to ensure a pragmatic and well-defined set of outcomes the results of the Delphi study will be discussed in an (international) expert meeting. The influence of this meeting on which outcomes are selected for the final COS is however very limited, as this is decided in the prior Delphi study.

**Involving parents and their children**

Involving patients in COS development has recently become common practice with 88% (n=112 as of April 12$^{th}$ 2016) of ongoing COS development studies doing so[18]. Involving patients as participants seems imperative as patients may identify different outcomes that should be measured, compared with physicians[16]. Several recent COS development projects have attempted to engage children/young people in developing their COS, either as part of the advisory group or the steering committee, or as a stakeholder group in the Delphi[15,22], focus groups[31], interviews[32] or as a part of the consensus meeting[33]. Attempts to engage children and young people in an online Delphi questionnaire have proven to be difficult. In the UK COS for uncomplicated appendicitis, there were substantial difficulties with retaining young people in the consecutive rounds of the Delphi questionnaire,



For peer review only - http://bmjopen.bmj.com/site/about/guidelines.xhtml

despite extensive efforts to optimize the methodology to appeal to children and young people, including: preliminary semi-structured interviews on the subject, pre-testing of the Delphi survey by young people and children[15] and video animations explaining the need for a COS. Parent participation however showed more promising results. Consequently, to safeguard the input of children/young people, the Delphi questionnaire for this study will be developed to be completed by parents with input from their children (5-18 years) whenever possible. In order to ensure that there are no large discrepancies between the opinions of parents with their children, and with children without their parents, we will organize a form of in-person interaction with young people (12-18 years) who have been treated for appendicitis. Involving children/young people in COS development is a subject of interest in many ongoing COS development projects. If experience from these groups warrants a change in our methodology, we will adjust our protocol accordingly during the course of the development and publish the updated protocol on an online, open source format (via the Open Science Framework).

A limitation is that due to the international nature of our study it will not be feasible to engage children directly in all the participating countries. That is why the face-to-face engagement will take place in selected countries.

**Other stakeholders**

After careful consideration and consultation with the participating countries, it was decided not to include paediatricians, general practitioners, or emergency medicine physicians. Although all these specialists play an intricate role in the diagnosis and care for children with appendicitis, they do not make the final decision regarding treatment or its provision. We will however, depending on the organisation of the healthcare system in each country, ask these stakeholders to comment on the final COS in order to ensure that essential outcomes are not missed. Since almost all research regarding treatment of paediatric uncomplicated appendicitis is initiated by (paediatric) surgeons, it was decided that researchers will not be included as a separate individual stakeholder group. However, involvement in research will be registered. Whilst their opinion is vital to the development of a COS, it is likely researchers will be well represented in the (paediatric) surgeon stakeholder group. A stratified analyses will be performed to check for skewing of the results by surgeons involved in research. It was also decided not to include journal editors or healthcare commissioners. Even though their opinion is of great importance especially regarding implementation, it was determined that their opinion is not essential in establishing the outcomes selected for the COS. There is much variability between countries regarding the role of these stakeholders, which would lead to major challenges regarding Delphi analyses of such a small stakeholder group. However, to enhance implementation and because of their expertise on the use of COSs,



For peer review only - http://bmjopen.bmj.com/site/about/guidelines.xhtml

475 representatives from these stakeholder groups will be asked to attend the final consensus
476 discussion.

477 **Outcome measures**

478 This study will not answer the question on how to measure the outcomes that are included in
479 the final COS, or at what time point the outcomes should be measured. We will however
480 attempt to come to a clear definition of each outcome. We expect that further research will be
481 necessary to answer the question of timing and how to measure the outcomes. We will
482 advise on this subject in the final report.

For peer review only



For peer review only - http://bmjopen.bmj.com/site/about/guidelines.xhtml

# FOOTNOTES

**Collaborators:**

The pediatric surgery departments of following hospitals have initiated the COS project and will contribute by recruiting participants. Nationwide Children's Hospital, Columbus, OH, USA. Hasbro Children's Hospital and Alpert Medical School of Brown University, Providence, RI, USA. Children's Mercy Hospital, Kansas City, MO, USA. Karolinska University Hospital, Stockholm, Sweden. Southampton General Hospital, Southampton, UK. Hôpital des Enfants, Centre Hospitalier Universitaire Toulouse, Toulouse, France. Hôpital Femme-Enfant, University Hospital, CHU Rennes, Rennes, France. Sydney Children’s Hospital, Randwick NSW, Australia. KK Women's and Children's Hospital, Singapore. BC Children’s Hospital, Vancouver, BC, Canada. The Hospital for Sick Children, Toronto, ON, Canada. Montreal Children’s Hospital, Montreal, QC, Canada. Children’s Hospital of Manitoba, Winnipeg, MB, Canada. Emma Children’s Hospital, Amsterdam UMC, University of Amsterdam, Vrije Universiteit, Amsterdam, Netherlands. Helsinki Children’s Hospital, Helsinki, Finland. Universiti Kebangsaan Malaysia Medical Centre (UKMMC), Kuala Lumpur, Malaysia.

**Acknowledgments**

We acknowledge Hester Rippen as the representative of the Dutch Foundation Children and Hospital for her advice and support in drafting the protocol.

**Author contributions:**

All authors have contributed to the design of this protocol. MK, NJH JHvdL, RB and RRG have initiated the project. The protocol was drafted by MK which was refined by NJH, JHvdL, RB, MO, NJB, LWEvH, and RRG. Statistical advice was provided by JHvdL. MK was responsible for drafting this manuscript. All authors have contributed to the manuscript and read and approved the final manuscript. The paediatric appendicitis COS development group consist of all local investigators who are responsible for translation, ethical board approval, participant recruitment. They have all read and approved the final manuscript.

**Data sharing statement:**

The project is registered on the comet-initiative.org which is open access. The study findings will be presented in a report which will be submitted for publication in a relevant peer-reviewed journal to ensure dissemination to relevant healthcare professionals. Findings may also be submitted for presentation at local meetings or conferences. The protocol will be published on an open access repository.

**Funding statement**: This research received no specific grant from any funding agency in the public, commercial or not-for-profit sectors

**Competing interests statement:** None to declare

For peer review only

# REFERENCES

1 Addiss DG, Shaffer N, Fowler BS, *et al.* the Epidemiology of Appendicitis and Appendectomy in the United States. *Am J Epidemiol* 1990;**132**:910–25. doi:10.1093/oxfordjournals.aje.a115734

2 Anderson JE, Bickler SW, Chang DC, *et al.* Examining a common disease with unknown etiology: trends in epidemiology and surgical management of appendicitis in California, 1995-2009. *World J Surg* 2012;**36**:2787–94. doi:10.1007/s00268-012-1749-z

3 Ferris M, Quan S, Kaplan BS, *et al.* The Global Incidence of Appendicitis. *Ann Surg* 2017;**266**:237–41. doi:10.1097/SLA.0000000000002188

4 Georgiou R, Eaton S, Stanton MP, *et al.* Efficacy and Safety of Nonoperative Treatment for Acute Appendicitis: A Meta-analysis. *Pediatrics* 2017;:e20163003. doi:10.1542/peds.2016-3003

5 Huang L, Yin Y, Yang L, *et al.* Comparison of Antibiotic Therapy and Appendectomy for Acute Uncomplicated Appendicitis in Children. *JAMA Pediatr* 2017;**171**:426. doi:10.1001/jamapediatrics.2017.0057

6 Xu J, Adams S, Liu YC, *et al.* Nonoperative management in children with early acute appendicitis: A systematic review. *J Pediatr Surg* Published Online First: May 2017. doi:10.1016/j.jpedsurg.2017.05.003

7 Kessler U, Mosbahi S, Walker B, *et al.* Conservative treatment versus surgery for uncomplicated appendicitis in children: a systematic review and meta-analysis. *Arch Dis Child* 2017;:archdischild-2017-313127. doi:10.1136/archdischild-2017-313127

8 Gorter RR, The S-MML, Gorter-Stam MAW, *et al.* Systematic review of nonoperative versus operative treatment of uncomplicated appendicitis. *J Pediatr Surg* 2017;**18 Apr 201**. doi:10.1016/j.jpedsurg.2017.04.005

9 Sinha I, Jones L, Smyth RL, *et al.* A Systematic Review of Studies That Aim to Determine Which Outcomes to Measure in Clinical Trials in Children. *PLoS Med* 2008;**5**:e96. doi:10.1371/journal.pmed.0050096

10 Kirkham JJ, Dwan KM, Altman DG, *et al.* The impact of outcome reporting bias in randomised controlled trials on a cohort of systematic reviews. *BMJ* 2010;**340**:c365.



For peer review only - http://bmjopen.bmj.com/site/about/guidelines.xhtml

11 Pogue J, Yusuf S. Overcoming the limitations of current meta-analysis of randomised controlled trials. *Lancet (London, England)* 1998;**351**:47–52. doi:10.1016/S0140-6736(97)08461-4

12 Zanchetti A, Mancia G. Searching for information from unreported trials--amnesty for the past and prospective meta-analyses for the future. *J Hypertens* 1998;**16**:125.

13 Hall NJ, Kapadia MZ, Eaton S, *et al.* Outcome reporting in randomised controlled trials and meta-analyses of appendicitis treatments in children: a systematic review. *Trials* 2015;**16**:275. doi:10.1186/s13063-015-0783-1

14 Clarke M. Standardising outcomes for clinical trials and systematic reviews. *Trials* 2007;**8**:39. doi:10.1186/1745-6215-8-39

15 Sherratt FC, Eaton S, Walker E, *et al.* Development of a core outcome set to determine the overall treatment success of acute uncomplicated appendicitis in children: a study protocol. *BMJ Paediatr Open* 2017;**1**:e000151. doi:10.1136/bmjpo-2017-000151

16 Sanderson T, Morris M, Calnan M, *et al.* What outcomes from pharmacologic treatments are important to people with rheumatoid arthritis? Creating the basis of a patient core set. *Arthritis Care Res (Hoboken)* 2010;**62**:640–6. doi:10.1002/acr.20034

17 Kirkham JJ, Davis K, Altman DG, *et al.* Core Outcome Set-STAndards for Development: The COS-STAD recommendations. *PLOS Med* 2017;**14**:e1002447. doi:10.1371/journal.pmed.1002447

18 Williamson PR, Altman DG, Bagley H, *et al.* The COMET Handbook: version 1.0. *Trials* 2017;**18**:280. doi:10.1186/s13063-017-1978-4

19 Kirkham JJ, Gorst S, Altman DG, *et al.* Core Outcome Set-STAndards for Reporting: The COS-STAR Statement. *PLoS Med* 2016;**13**:e1002148. doi:10.1371/journal.pmed.1002148

20 Staniszewska S, Brett J, Simera I, *et al.* GRIPP2 reporting checklists: tools to improve reporting of patient and public involvement in research. *BMJ* 2017;**358**:j3453.

21 Protocol for the development of a global core outcome set for treatment of uncomplicated appendicitis in children :: Core Outcome Measures in Effectiveness Trials Initiative (COMET). http://www.comet-initiative.org/studies/details/1119 (accessed 5 Mar 2018).

22 Sinha IP, Smyth RL, Williamson PR. Using the Delphi Technique to Determine Which

For peer review only



For peer review only - http://bmjopen.bmj.com/site/about/guidelines.xhtml

Outcomes to Measure in Clinical Trials: Recommendations for the Future Based on a Systematic Review of Existing Studies. *PLoS Med* 2011;**8**:e1000393. doi:10.1371/journal.pmed.1000393

23 Liberati A, Altman DG, Tetzlaff J, *et al.* The PRISMA statement for reporting systematic reviews and meta-analyses of studies that evaluate health care interventions: explanation and elaboration. *J Clin Epidemiol* 2009;**62**:e1–34. doi:10.1016/j.jclinepi.2009.06.006

24 Boers M, Kirwan JR, Gossec L, *et al.* How to choose core outcome measurement sets for clinical trials: OMERACT 11 approves filter 2.0. *J Rheumatol* 2014;**41**:1025–30. doi:10.3899/jrheum.131314

25 Mullen PM. Delphi: myths and reality. *J Health Organ Manag* 2003;**17**:37–52. doi:10.1108/14777260310469319

26 Murphy MK, Black NA, Lamping DL, *et al.* Consensus development methods, and their use in clinical guideline development. *Health Technol Assess* 1998;**2**:i–iv, 1-88.

27 COMET DelphiManager. 2017.http://www.comet-initiative.org/delphimanager/ (accessed 4 Dec 2018).

28 Guyatt GH, Oxman AD, Kunz R, *et al.* GRADE guidelines: 2. Framing the question and deciding on important outcomes. *J Clin Epidemiol* 2011;**64**:395–400. doi:10.1016/j.jclinepi.2010.09.012

29 Brookes ST, Macefield RC, Williamson PR, *et al.* Three nested randomized controlled trials of peer-only or multiple stakeholder group feedback within Delphi surveys during core outcome and information set development. *Trials* 2016;**17**:409. doi:10.1186/s13063-016-1479-x

30 Biggane AM, Brading L, Ravaud P, *et al.* Survey indicated that core outcome set development is increasingly including patients, being conducted internationally and using Delphi surveys. *Trials* 2018;**19**:113. doi:10.1186/s13063-018-2493-y

31 Tsichlaki A, O'Brien K, Johal A, *et al.* Development of a core outcome set for orthodontic trials using a mixed-methods approach: protocol for a multicentre study. *Trials* 2017;**18**:366. doi:10.1186/s13063-017-2098-x

32 Allard A, Fellowes A, Shilling V, *et al.* Key health outcomes for children and young people with neurodisability: qualitative research with young people and parents. *BMJ Open* 2014;**4**:e004611. doi:10.1136/bmjopen-2013-004611



For peer review only - http://bmjopen.bmj.com/site/about/guidelines.xhtml

33 Morris C, Janssens A, Shilling V, *et al.* Meaningful health outcomes for paediatric neurodisability: Stakeholder prioritisation and appropriateness of patient reported outcome measures. *Health Qual Life Outcomes* 2015;**13**:87. doi:10.1186/s12955-015-0284-7

For peer review only

**Online supplement 1.**

S1. Completed COS-STAD checklist for PA-COS project

| Domain | Methodology | Notes | Addressed on page number |
|---|---|---|---|
| Scope specification | 1. The research or practice setting(s) in which the COS is to be applied | e.g., for application in research studies or for use in routine care | 4. |
| | 2. The health condition(s) covered by the COS | e.g., treatment of rheumatoid arthritis or screening for cancer | 4. |
| | 3. The population(s) covered by the COS | e.g., patients with advanced disease or children | 4. |
| | 4. The intervention(s) covered by the COS | e.g., all interventions, drug therapy, or surgical interventions | 4. |
| Stakeholders involved | 5. Those who will use the COS in research | e.g., clinical trialists or industry | 6. |
| | 6. Healthcare professionals with experience of patients with the condition | e.g., clinical experts, practitioners, and investigators with particular experience in the condition | 6. |
| | 7. Patients with the condition or their representatives | involve those who have experienced or who are affected by the condition (e.g., patients, family members, and carers). | 6. |
| Consensus process | 8. The initial list of outcomes considered both healthcare professionals' and patients' views. | consider the views of healthcare professionals and patients (most likely identified from literature reviews or interviews) when generating an initial list of outcomes for inclusion in the consensus process. | 5. (No patient involvement) |
| | 9. A scoring process and consensus definition were described a priori. | Although different consensus methods may be employed in different studies, to avoid any potential biases, COS developers should describe their consensus method a priori. | 7, 8, 9. |
| | 10. Criteria for including/dropping/adding | prespecify criteria for including, dropping, or adding new outcomes to avoid potential biases. | 7, 8, 9. |
| | 11. Care was taken to avoid ambiguity of language used in the list of outcomes. | consider the language used when describing outcomes in front of different stakeholder groups. An example of 1 approach taken is to include both lay and medical terms, with these previously piloted with the stakeholders. | 7,8. |

For peer review only

**Online supplement 2.**

S2. Search strategy for systematic review PA-COS

**Appendicitis**

“Appendix"[Mesh] OR appendix[tiab] OR appendix[ot] OR "Appendicitis"[Mesh] OR "Appendectomy"[Mesh] OR appendicit*[tiab] OR appendicit*[ot] OR appendectom*[tiab] OR appendectom*[ot] OR appendicectom*[tiab] OR appendicectom*[ot]

**Children**

child*[tw] OR schoolchild*[tw] OR infan*[tw] OR adolescen*[tw] OR pediatri*[tw] OR paediatr*[tw] OR neonat*[tw] OR boy[tw] OR boys[tw] OR boyhood[tw] OR girl[tw] OR girls[tw] OR girlhood[tw] OR youth[tw] OR youths[tw] OR baby[tw] OR babies[tw] OR toddler*[tw] OR teen[tw] OR teens[tw] OR teenager*[tw] OR newborn*[tw] OR postneonat*[tw] OR postnat*[tw] OR perinat*[tw] OR puberty[tw] OR preschool*[tw] OR suckling*[tw] OR picu[tw] OR nicu[tw]

**3.2.2 Study selection**

Selection of studies will be performed by 2 independent reviewers (MK, JF) according to the below mentioned in- and exclusion criteria. In case of disagreement between two reviewers, a third independent reviewer (RG) will make the final decision.

*Inclusion/Exclusion Criteria:*

All RCTs and systematic reviews/meta-analyses reporting the outcome of treatment of acute uncomplicated appendicitis will be included in this systematic review. By including systematic reviews that also report on non-comparative studies we expect to identify all reported treatment outcomes, including those from the relatively new field of non-operative management of uncomplicated appendicitis. Publications before January 2014 will be excluded. Only studies in children (<18 years of age) will be included. Studies only reporting on the outcome of treatment in complex or complicated appendicitis (gangrenous appendicitis, appendiceal mass, appendiceal abscess) will be excluded.

**3.2.3 Data extraction**

The two reviewers will extract the data independently using the predefined data extraction form shown in Appendix A. In case of disagreement a third reviewer will make the final decision. A risk of bias assessment of the individual studies is not applicable as we will not be using individual study data but only the reported outcomes. As diversity in terminology will be anticipated, we decided to initially report all outcome measure as mentioned in the original study. Outcome measures will be mapped independently by two reviewers and in case of disagreement a third reviewer will make the final decision. After data extraction of all studies is completed, a meeting of the study management group will be held between to discuss potential similarity between the outcome measures in order to assign an appropriate term for them.



For peer review only - http://bmjopen.bmj.com/site/about/guidelines.xhtml

# Establishing a core outcome set for treatment of uncomplicated appendicitis in children: study protocol for an international Delphi survey.

| | |
|---|---|
| Journal: | *BMJ Open* |
| Manuscript ID | bmjopen-2018-028861.R1 |
| Article Type: | Protocol |
| Date Submitted by the Author: | 11-Mar-2019 |
| Complete List of Authors: | Knaapen, Max; Emma Children's Hospital, Amsterdam UMC, University of Amsterdam, Vrije Universiteit, Paediatric Surgical Centre<br>Hall, Nigel; University of Southampton Faculty of Medicine, University Surgery Unit<br>van der Lee, Johanna H.; Emma Children's Hospital, Amsterdam UMC, University of Amsterdam, Paediatric clinical Research Office<br>Butcher, Nancy; Sick Kids, Child Health Evaluative Sciences<br>Offringa, Martin; The Hospital for Sick Children, University of Toronto, Department of Paediatrics<br>Van Heurn, Ernst; Emma Children's Hospital, Amsterdam UMC, University of Amsterdam, Vrije Universiteit, Paediatric Surgical Centre<br>Bakx, Roel; Emma Children's Hospital, Amsterdam UMC, University of Amsterdam, Vrije Universiteit, Paediatric Surgical Centre<br>Gorter, Ramon; Emma Children's Hospital, Amsterdam UMC, University of Amsterdam, Vrije Universiteit, Paediatric Surgical Centre<br>+ On behalf of the paediatric , appendicitis COS development group |
| <b>Primary Subject Heading</b>: | Surgery |
| Secondary Subject Heading: | Emergency medicine, Gastroenterology and hepatology, Medical publishing and peer review, Paediatrics, Research methods |
| Keywords: | delphi, appendicitis, children, core outcome set, paediatric, Global |
| | |

SCHOLARONE™
Manuscripts

# Establishing a core outcome set for treatment of uncomplicated appendicitis in children: study protocol for an international Delphi survey.

Knaapen M, MD[1], Hall NJ, MD, PhD[2], Van der Lee JH, MD, PhD[3], Butcher NJ, PhD[4], Offringa M, MD, PhD[4, 5], Van Heurn, Ernst W.E, PhD[1], Bakx R, MD, PhD[1], Gorter RR, MD, PhD[1] + On behalf of the paediatric appendicitis COS development group

1. Emma Children's Hospital, Amsterdam UMC, University of Amsterdam & Vrije Universiteit, Department of Paediatric Surgery, Amsterdam, The Netherlands. Electronic address: rr.gorter@vumc.nl / r.bakx@amc.uva.nl / m.knaapen@uva.amc.nl / e.vanheurn@amc.uva.nl
2. University Surgery Unit, Faculty of Medicine, University of Southampton, Southampton General Hospital, Southampton, UK; Department of Paediatric Surgery and Urology, Southampton Children's Hospital, Southampton, UK. Electronic address: n.j.hall@soton.ac.uk
3. Emma Children's Hospital, Amsterdam UMC, University of Amsterdam, Paediatric clinical Research Office, Amsterdam, The Netherlands. Electronic address: j.h.vanderlee@amc.uva.nl
4. Child Health Evaluative Sciences, The Hospital for Sick Children Research Institute, Toronto, Canada; Electronic address: nancy.butcher@sickkids.ca
5. Department of Paediatrics, The Hospital for Sick Children, University of Toronto, Toronto, Canada. Electronic address: martin.offringa@sickkids.ca

Address correspondence to Max Knaapen, MD

Address: Emma Children's Hospital, Amsterdam UMC, University of Amsterdam, Vrije Universiteit, Paediatric Surgical Centre, Amsterdam. PO Box 22660, 1100DD Amsterdam, the Netherlands.

Email: M.Knaapen@AMC.UvA.nl

Telephone: +31-20-5665693

Word count: 5753

Funding statement: None

Competing interests statement: None to declare



For peer review only - http://bmjopen.bmj.com/site/about/guidelines.xhtml

# ABSTRACT

**Introduction**

Appendicitis is a global disease affecting roughly one in every 12 people in the world, with the highest incidence between ages 10 and 19 years. To date, a wide variety of health outcomes have been reported in randomized controlled trials (RCTs) and meta-analyses evaluating treatments for appendicitis. This is especially the case in studies comparing non-operative treatment to operative treatment. A set of standard outcomes, to be reported in all future trials, is needed to allow for adequate comparison and interpretation of clinical trial results and to make data pooling possible. This protocol describes the development of such a global core outcome set (COS) to allow unified reporting of treatment interventions in children with acute uncomplicated appendicitis.

**Methods and analysis**

We use current international standard methodology for the development and reporting of this COS. Its development consists of three phases: (1) Update the most recent systematic review on outcomes reported in uncomplicated paediatric appendicitis research, to identify additional outcomes, (2) Three-step global Delphi study to identify a set of core outcomes for which there is consensus between parents and (paediatric) surgeons, and (3) Expert meeting to finalize the COS and its definitions. Children and young people will be involved through their parents during phase two and will be engaged directly using a customized face-to-face approach.

**Ethics and dissemination.**

The medical research ethics committee of the Academic Medical Centre Amsterdam has approved the study. Each participating country/research group will ascertain ethics board approval. Electronic informed consent will be obtained from all participants. Results will be presented in peer-reviewed academic journals and at (international) conferences.

**Registration details**

The COS development project was registered with the COMET initiative in February 2018 (http://www.comet-initiative.org/studies/details/1119).


## Article Summary: Strengths and limitations of this study

1. A globally relevant set of core outcomes for paediatric uncomplicated appendicitis assessed in an international online Delhi study.
2.A that protocol was developed in conjunction with an international steering committee, including patient representation.
3. Involvement of parents and their children in choosing what to measure in future uncomplicated appendicitis research.
4. Direct face-to-face involvement of young people, however, only in selected countries.
5. A limited consensus discussion with only selected individuals.



For peer review only - http://bmjopen.bmj.com/site/about/guidelines.xhtml

# INTRODUCTION

Appendicitis is a common gastro-intestinal disease affecting roughly one in every 12 people in the world, with the highest incidence between ages 10 and 19 years[1,2]. While the incidence varies from country to country, appendicitis is a global disease[3]. In the last decade, there have been several developments in the treatment of appendicitis in children, with the most recent being non-operative treatment (NOT) for acute uncomplicated appendicitis. Studies investigating the effectiveness of NOT in children show promising results[4–7]. However, the selected primary (and secondary) outcomes vary widely, as reflected in recent systematic reviews assessing the efficacy and safety of NOT, this may contribute to their contradictory conclusions[4–8]. In the systematic review by Georgiou et al.[4], the need for universal outcome selection and reporting in appendicitis studies is emphasized. In general, it is recognized that clinical trials in children often lack outcomes that are appropriately chosen for this particular population[9].

Inconsistent selection and reporting of outcomes limits the ability to adequately compare and interpret clinical trial results. Furthermore, it hampers data pooling and subsequent meta-analysis. It also increases the risk of selective outcome reporting, a form of publication bias. This in turn jeopardizes the validity of results from individual trials, which feeds into subsequent systematic reviews[10] and meta-analyses, which are by nature retrospective, and therefore liable to various risks of bias[11,12].

As demonstrated by Hall et al. in 2015, a wide variety of outcomes has been reported in randomized controlled trials (RCTs) and meta-analyses reporting on the treatment of appendicitis in children[13]. In the 63 included studies, a total of 115 different outcomes were reported[13]. Hall et al. proposed the development of a Core Outcome Set (COS), which is a standardized collection of outcomes that should be measured and reported in all future trials[14]. Recently a study protocol was published for developing such a COS in the United Kingdom[15]. To overcome any limitations of a COS focused on UK-specific surgical practice, the aim of this study is to develop an international COS to be used for trials assessing the treatment of acute uncomplicated appendicitis in children. The development of this protocol and the international COS is being performed in conjunction with the UK research group. The principal investigator of UK COS (NJ Hall) has been involved from the beginning of the protocol development and is part of the study management group. Outcomes considered important by patients and families are essential to a meaningful and complete COS[16]. That is why parents and patients will play a central role in the consensus process as a stakeholder group. Parent and patient representation was insured through involvement of the Dutch patient and parent Foundation: “Children and Hospital”. A representative from this group provided feedback from the perspective of parents and



For peer review only - http://bmjopen.bmj.com/site/about/guidelines.xhtml

children in several stages of the protocol development. They will also be involved in the development of a face-to-face methodology for engaging children in this COS project.

## Scope

We aim to reach a global consensus amongst patients, parents, researchers and physicians on the minimal set of core outcomes that should be measured and reported in all future clinical trials investigating any type of treatment for acute uncomplicated appendicitis in children, including surgical treatment, non-operative treatment or other treatment aimed at curing appendicitis.

# METHODS

In the development of this protocol, we adhere to the COS-STAD (Core Outcome Set-STAndards for Development) recommendations[17] and the COMET (Core Outcome Measures in Effectiveness Trials) handbook[18]. The completed COS-STAD checklist can be found in online supplement S1. The final core outcome set will be reported in accordance with the COS-Standards for reporting (COS-STAR) statement[19]. Involvement of patients and the public will be described using the GRIPP2 reporting checklist[20] (Guidance for Reporting on Involvement of Patients and Public). This study was registered with the COMET initiative (registration number: 1119) on February 11, 2018[21].

### Study design

The paediatric appendicitis COS (PA-COS) development will consist of three phases: (1) An update of the 2015 systematic review on outcomes reported in uncomplicated paediatric appendicitis research[13], in order to identify any additional outcomes used in trials that were published since the previous systematic review; (2) A three-step Delphi study to identify a set of core outcomes from those selected in the literature review. Development of the Delphi is performed according to the checklist by Sinha et al.[22] on the design and reporting of Delphi studies concerning COS selection; and (3) An expert panel meeting to ratify the final COS, including physicians, researchers and children/parent representatives. Children and young people will be involved through their parents during phase two and will be engaged directly using a customized approach.

### Steering Committee

An international steering committee has been established and consists of the following; the authors, a parent/patient representative of the Dutch Foundation: “Children and Hospital”, and the lead local investigator of each participating centre (paediatric appendicitis COS



For peer review only - http://bmjopen.bmj.com/site/about/guidelines.xhtml

development group). The steering committee will agree on the final version of the protocol at the start of the project and will provide input throughout the duration of the project. The steering committee members will also be involved in the development of the final COS. Within the steering committee, a smaller study management group has been appointed which will convene during regular (videoconference) meetings.

## Systematic review: Treatment outcomes

Hall et al. performed a systematic review of RCTs and meta-analyses reporting treatment outcomes in children with appendicitis up to April 2014[13]. They found a 115 unique outcomes which they collapsed into a total of 38 standardized outcome terms. We will update the systematic review to identify any new unique outcomes in clinical trials or systematic reviews. All RCTs and systematic reviews/meta-analyses reporting treatment outcomes of acute uncomplicated appendicitis in children (<18 years of age) published between January 1st 2014 and November 23th 2017 will be included. The final review will follow the PRISMA reporting guideline[23]. We will search the Cochrane Central Register of Controlled Trials, MEDLINE and EMBASE with the help of a clinical librarian. Additional information on the search strategy/study selection and data extraction can be found in online supplement S2. Studies only reporting outcomes of treatment in complex or complicated appendicitis (for example - gangrenous or perforated appendicitis, appendiceal mass, appendiceal abscess) will be excluded.

After data extraction, a meeting of the study management group (including NJ Hall) will be held to discuss potential similarities between the outcomes from the 2015 systematic review from Hall et al.[13]. New unique outcomes will be discussed within the group in order to assign an appropriate standardized outcome term. If these outcomes do not match any of the original 38 outcome terms a new term will be assigned, figure 1. illustrates this methodology. The new and original outcome terms will be mapped to four core areas (death, life impact, resource use, pathophysiological manifestations) in accordance with the methods from the OMERACT FILTER 2.0[24]. Although Hall et al.[13] chose to list the adverse events as a separate core area, we will reclassify these outcome terms to one of the four core areas (Table 1.). We will however, label adverse events of treatment separately, as the OMERACT filter suggests[24]. A meeting of the study management group will be held to discuss potential similarity between outcomes and to assign an appropriate outcome term for similar outcomes. Outcomes that are only found once and are not generalizable can be excluded (e.g. the width of lateral thermal damage of the mesoappendix after appendectomy). Grouping the outcomes under a common outcome term aims to arrive at a manageable and cohesive list of outcomes that is appropriate as a basis for the Delphi questionnaire.



For peer review only - http://bmjopen.bmj.com/site/about/guidelines.xhtml

For peer review only

**Table 1. Outcome Core Areas**

| Core Area | Example(s) |
|---|---|
| Life impact | *Quality of life, loss of ability to work* |
| Resource use | *Length of hospital stay, healthcare costs, societal cost* |
| Pathophysiological Manifestations | *Biochemical parameters, organ function, (ir)reversible manifestations (complications, pathology results)* |
| Death | *Death* |

## Stakeholders and recruitment

1) **Children and Young People**

Children and young people (5-18 years) who have been treated for acute uncomplicated appendicitis in the preceding 24 months, either with initial NOT or with surgery. Children less than 5 years old are excluded since different outcomes might be appropriate in this very young age group. Also, uncomplicated appendicitis is much less common in young children than in older children. Furthermore, there are no studies in which children below the age of 5 are treated non-operatively. Children will be engaged indirectly as we will urge parents to discuss the answers they provide with their child whilst filling out the Delphi questionnaire. Young people will be engaged directly through a customized face-to-face approach in selected countries. For the invited children, considering the complexity of the subject and methodology, age is limited to 12-18 years.

2) **Parents**

Parents of children and young people (5-18 years) treated for acute uncomplicated appendicitis either with initial NOT or with surgery in the preceding 24 months or during the initial phase of the study. Parents will be asked to discuss the answers they provide with their child whilst filling out the Delphi questionnaire. Parents will be invited to participate by their child's treating physician or their designate in each



For peer review only - http://bmjopen.bmj.com/site/about/guidelines.xhtml

participating country/hospital. Participants will be identified retrospectively by contacting patients that were treated in the past 24 months or prospectively by inviting patients to participate directly after they have completed their treatment.

3) **(Paediatric) Surgeons**

General and/or paediatric surgeons who care for children in the specified age group will be asked to participate. Surgeons will be identified and invited by the local coordinators in each participating country. These local coordinators are research groups that have previously registered a clinical trial on uncomplicated appendicitis in children. This should allow for inclusion of physicians that also have experience in research on the treatment of appendicitis.

**Participating countries and research groups**

It was decided to invite research groups that are currently conducting clinical trials on the treatment of acute uncomplicated in children. Groups were identified through www.clinicaltrials.gov by searching (January 2017) for 'appendicitis' with an age limitation of 5-18 years. Studies with a mixed population (children and adults) were excluded. Studies that had been completed before 2014, had not been updated since 2015, or with incomplete registrations, were excluded. We found 111 trials, of which 12 trials assessed the treatment of uncomplicated appendicitis in children. Groups from the Netherlands, USA, Canada, Australia, Sweden, Finland, UK, France, Italy, Israel, Japan, Singapore and Malaysia were identified. Some trials included hospitals from multiple countries.

## Sample size

There is no rationale for determining the number of respondents to invite for a Delphi study[18]. A minimum of seven respondents per stakeholder group is suggested to have a large enough group to allow for a consensus process[25]. Taking into account that only some invited participants will register for the Delphi and that not all respondents will complete all rounds of the Delphi (attrition), a minimum of 40 respondents per stakeholder group per country will be invited. There will be no maximum. In case the number of respondents per country is significantly higher than other countries, we will consider a weightage per country in the analyses. We anticipate that this sample will be large enough to reflect all relevant opinions.

## Delphi study

International online Delphi study

The Delphi method is an effective tool for reaching consensus in a large group without the



For peer review only - http://bmjopen.bmj.com/site/about/guidelines.xhtml

need for face-to-face contact[26]. The use of sequential questionnaires which are answered anonymously by stakeholders is an established method for reaching consensus in a group of experts[22]. Questionnaires will be sent using DelphiManager[27], a web-based system designed for Delphi studies. The questionnaires will be open simultaneously to all respondents of the participating countries. After each round, the responses of all participants are shared anonymously in accordance with the Delphi principle.

The list of outcomes from the systematic review will be formatted into questions accompanied by an extensive plain language summary per outcome, including figures if appropriate. The Delphi questionnaire will originally be formulated in English and will be translated if required. Translation will only be performed by native speaking professionals.

Participants will be asked to score the importance of each outcome using a 1 to 9 Likert scale as recommended by the Grading of Recommendations Assessment, Development and Evaluation (GRADE) working group[28] and COMET initiative[18]. A score of 7-9 indicates a critical outcome for assessing the effect of a treatment, 4-6 important but not critical, 1-3 indicates an outcome with low importance for assessing the treatment effect. It will also be possible to select an “unable to score” option, which is especially of importance if parents do not feel equipped to score certain outcomes. The questionnaires including the plain language summaries will be piloted by a group of laypersons (n=10) to check for ambiguity and readability.

Delphi round one

Participants will be divided into two stakeholder groups: parents (with their children), and surgeons. Parents will be asked to discuss the answers they provide with their child whilst filling out the Delphi questionnaire. Baseline characteristics (age, country) will be ascertained. Parents will be asked if their child was treated with non-operative or operative treatment, time between registration and the first diagnosis of appendicitis and if their treatment was with or without complications. And also if they will be answering the Delphi whilst consulting their child. Surgeons will be asked their speciality (paediatric, general, abdominal, other), workplace (academic, teaching hospital, non-teaching hospital), experience with non-operative treatment, experience in research regarding appendicitis in children.

All participants will be asked to score all previously identified outcomes according to their perceived importance for assessing the treatment effect. In the first round there will be an option to suggest additional outcomes not yet listed.

Participants will have between four and eight weeks to complete each round, depending on the response rate. In that time they will receive a reminder email every two weeks as long as

For peer review only

they have not replied to the questionnaire.

Delphi round one: analysis

Results will be analysed **by stakeholder group and for all participants** using descriptive statistics. Outcomes will be analysed separately for each stakeholder group, as there is evidence that patients are likely to assign importance to outcomes differently than surgeons[29], which has the potential to influence eventual outcome selection.

"Consensus-in" will be defined as:

– Greater than 70% of participants in **both** stakeholder groups scoring the outcome as 7-9 and less than 15% in **both** stakeholder groups scoring the outcome as 1-3.

– Greater than 90% of participants within **one** stakeholder group scoring the outcome as 7-9. This implies that these outcomes are highly regarded by an individual stakeholder group, and should also be included[18].

"Consensus-out" will be defined as:

– Greater than 70% of participants in **both** stakeholder groups scoring the outcomes as 1-3 and less than 15% of participants in **both** stakeholder groups scoring the outcome as 7–9. Consensus-out can only be reached when there is consensus across **both** stakeholders groups.

Outcomes that do not meet any of these criteria will be defined as "no consensus". A stratified analysis will be performed to check for skewing as a result of divergent opinions from a single country or surgeons with or without research experience.

At the end of round one, there will be a meeting of the study management group to assess whether an alteration in the Delphi study is appropriate. If additional outcomes are suggested by Delphi participants, each outcome will be assessed by the study management group to determine whether it is indeed new and to which category it should be classified. Wording of the Delphi questionnaire will be adjusted if misinterpretation is suspected.

Delphi rounds two and three

All participants who have completed the previous round will be asked to participate in the next round. Only outcomes that have not been defined as "consensus-in" or "consensus-out" during the previous round will be presented in the following rounds to all participants. Outcomes for which there was "consensus-in" only within a single stakeholder group, will still be presented to the other stakeholder group to evaluate whether consensus can be achieved in both stakeholder groups. An overview of included and excluded outcomes will be



For peer review only - http://bmjopen.bmj.com/site/about/guidelines.xhtml

available. The outcomes for which there is no consensus and the newly suggested outcomes from the previous rounds will be presented with the participants' individual score and the median scores from each stakeholder group combined with a histogram showing the scoring distribution. Participants will be asked again to score all remaining outcomes in the same manner as in round one.

Delphi rounds two and three analysis

Results will be analysed per stakeholder group and for all participants, using descriptive statistics with the same definitions for consensus in/out as in the first Delphi round, including a stratified analysis. After the second round, there will be a meeting of the study management group to assess the need for alteration in the Delphi study, and to decide whether or not to proceed with a third Delphi round, assuming consensus between **both** stakeholder groups on more than 80% of the outcomes, and more than five outcomes with consensus in. To give an estimate of the degree of agreement between respondents, the width of the interquartile range of the median ranking score will be calculated, potentially ranging from 0.00, meaning complete agreement, to 8.00, meaning least possible agreement. This will be calculated for both the individual stakeholder groups as well as the entire group of respondents after the final round.

## Face-to-face engagement of young people

We wish to check for discrepancies of opinion between parents answering the Delphi together with their child and children who are interviewed directly. For this, a form of in-person interaction will be organised with young people (12-18 years) who have been treated for appendicitis. They will be asked to comment on the preliminary COS selection established at the end of the Delphi study, and to suggest additional outcomes and comment on outcomes that did not make the preliminary COS selection. This will either be done by a short, face-to-face, one round questionnaire involving only outcomes relevant to children/young people, or in the form of a small consensus meeting (prioritization meeting) before finalizing the definitive COS. Doing this type of research requires experienced interviewers and resources. That is why the face-to-face engagement will only take place in selected countries, however, we will aim to involve as many countries as feasible. Separate ethical board approval will be obtained as appropriate.

## Consensus discussion

If adequate consensus (we aim to achieve consensus on at least one outcome per OMERACT core area) is reached in the Delphi study, we will organise a face-to-face expert panel meeting with selected individuals with the purpose to ratify a pragmatic and well-defined set of outcomes. A secondary aim of this meeting is to enhance support and



For peer review only - http://bmjopen.bmj.com/site/about/guidelines.xhtml

implementation of the final COS.

The meeting will be held at an international conference for paediatric surgery. Through purposive sampling, approximately 30 “experts” from across all stakeholder groups, including physicians, researchers and children/parent representatives, will be invited to participate in a face-to-face meeting with the Steering Committee. Journal editors and healthcare commissioners will also be invited to attend in an observational capacity with the purpose of promoting implementation and to provide comments on the final list of outcomes.

In the event that adequate consensus cannot be reached in the Delphi process, we will organise a formal face-to-face consensus meeting or teleconference. In that case, we will select an appropriate representation of all stakeholder groups from the panel members that participated in the Delphi study.

## Final COS development

The goal is to achieve a pragmatic COS that is applicable and feasible for all future trials that evaluate the treatment of uncomplicated appendicitis in children. There is no recommended maximum number of outcomes that should be included in a COS. However, if the final COS includes to many outcomes, the COS would not be feasible to use in practice. To achieve the goal of a pragmatic COS we aim to arrive at a maximum of 10 outcomes, the same maximum number as the UK COS protocol specifies[15]. As a minimum, we aim to have at least one outcome per core area. If the number of outcomes for which consensus is achieved greatly exceeds 10 outcomes, the outcomes with the highest level of consensus will be considered part of the suggested COS. We will however report all outcomes for which consensus is achieved. The highest level of consensus depends on whether there is consensus in both stakeholder groups, the median score that was appointed to the outcome, and the interquartile range of the median score as an estimate of the degree of consensus.

Only outcomes for which consensus is reached internationally will be selected. To test for country bias, stratified analyses of the Delphi results will be performed. The results from the face-to-face engagement of young people will be taken into account for the final COS selection and will be reported separately. If there is no consensus between patients, parents and healthcare professionals, an outcome can still be selected if there is clear consensus within a single stakeholder group. These will be reported separately. The final COS will be categorised according to the four core areas of the OMERACT filter[24]. We will also annotate the outcomes according to the recently published outcome taxonomy to maximise future data harmonisation[30].

## Patient and Public Involvement



For peer review only - http://bmjopen.bmj.com/site/about/guidelines.xhtml

Patient involvement is at the core of this study design. By asking parents and patients with experience in having simple appendicitis what outcomes they feel should be part of future research. To ensure our design is appropriate for parents and children we have involved the Dutch child and parents representation group as part of our steering committee. In that capacities they provide input on the protocol and study. To make sure the Delphi questionnaire is understandable and has no ambiguities it is checked by a group of laypersons before the start of the Delphi study. Part of the Delphi study is giving feedback to all its participants after each round, this will also be done with the final study results.

## Ethics and dissemination.

Ethics

The medical research ethics committee of the Academic Medical Centre Amsterdam confirmed that the Dutch Medical Research Involving Human Subjects Act (WMO) does not apply to this study and that complete approval of this study by the committee is not required. Each participating country/research group will be asked to obtain ethics board approval or confirm that ethics board approval it not required. Electronic informed consent will be obtained from all participants. The face-to-face engagement of young people (12-18 years) will take place in selected countries and separate ethics board approval will be obtained, as appropriate.

Data collection and confidentiality

All data will be handled confidentially and in accordance with the Dutch Personal Data Protection Act and the European General Data Protection Regulation (GDPR). DelphiManager[27] will be used for the online questionnaire. After informed consent from all participants only limited identifying information (name, email) will be ascertained during registration. This information will be stored separately from the answers given in the questionnaire and will only be used for the purpose of direct feedback and reminder emails. Access to personally identifiable data will be strictly limited.

Study status and dissemination

In the first quarter (Q1) of 2018 the following 13 countries were invited to participate in the project; Netherlands, USA, Canada, Australia, Sweden, Finland, UK, France, Italy, Israel, Japan, Singapore and Malaysia. Ten countries replied, Italy, Israel and Japan did not. In Q1 2018 the systematic review was finished. In Q2 2018 the Delphi questionnaire was developed and piloted. In Q3 2018 all materials were translated. Between Q4 2018 and Q1 2019 IRB applications were submitted in 10 countries and 15 participating centres. The anticipated start of the online Delphi study is May 2019. We anticipate to have the final COS ready by Q1 2020. Dissemination of the results will be accomplished by publication in an



For peer review only - http://bmjopen.bmj.com/site/about/guidelines.xhtml

For peer review only

international peer-reviewed scientific journal and by presentations at (international) conferences. By involving the majority of the principal investigators who are currently involved in research on uncomplicated appendicitis in children, we aim to optimize uptake of the final COS. By involving journal editors and healthcare commissioners in the face-to-face consensus discussion, we aim to ultimately have the COS introduced as a requirement in future outcome reporting on the treatment of uncomplicated appendicitis in children. We will also actively send out the final COS to relevant journal editors and funding bodies to promote uptake in future research.

# DISCUSSION

## Strengths and limitations of this study

### Outcomes selection

The selection of potential outcomes will be done systematically and will provide a selection for the first Delphi questionnaire that reflects most issues pertinent to the treatment of uncomplicated appendicitis. By including systematic reviews/meta-analyses that also report on non-comparative studies, we expect to identify all reported treatment outcomes, including those from the relatively new field of NOT for uncomplicated appendicitis.

To be able to arrive at a manageable list of outcomes that is appropriate for a Delphi study, the number of outcome terms needs to be somewhat limited. In order to achieve this, the outcomes derived from our systematic review will be merged in case of similarity. If outcomes are not generalizable and only reported once, they will be excluded. This will be proposed and prepared by two independent reviewers and discussed in the study management group. However, the merging of outcomes will inevitably lead to some loss of detail.

### Global consensus

In order to reflect the views of different stakeholders, a variety of groups will be part of the development of this COS. This is not only the case on a national level, but also on an international level, related to, for example, differences between countries in resources, treatment practises for acute uncomplicated appendicitis, and cultural differences. For example there is a large difference with regard to the standard length of hospital stay after an appendectomy for simple appendicitis. In the USA much effort is devoted to reduce the number of admission days, in the UK there is only limited attention for the duration of admission and for instance in Japan an admission for 5 days is not uncommon. We can expect that these kind of differences result in different opinions regarding the core outcomes set. By also involving patients and parents from the participating countries we hope to correct for these differences[31]. In conjunction with the UK paediatric appendicitis COS research



For peer review only - http://bmjopen.bmj.com/site/about/guidelines.xhtml

group we decided that an international validation of the UK COS would not give the depth of information and would not allow for consensus formation on all possible outcomes. Which we feel is appropriate considering the before mentioned significant differences between countries. Involving members from different countries will not only lead to the development of a COS that reflects the opinions of the international community, it should also lead to an internationally applicable “minimal” COS. However, selecting the participating countries on the basis of their involvement in research on appendicitis in children is a limitation. This choice was made on the basis of feasibility. Researchers in the field of uncomplicated appendicitis have an interest in the development of a COS and have the network to help carry out the Delphi study. With our current selection we will, however, have participants from four different continents. Our means of selection has another advantage. Since non-operative treatment is an important research subject in childhood appendicitis, we aim to include surgeons and parents who have experience in that field. As non-operative treatment is still experimental in most of the world, we need surgeons and patients who have been involved in such research.

**Limited face-to-face consensus**

If consensus is reached in the Delphi study we will not be organising a formal consensus meeting. The Delphi method can be used for reaching consensus in a group of respondents without the need for face-to-face contact. There is a risk of bias if a face-to-face consensus meeting leads to selection of only participants who are able to attend the meeting, which is especially a problem in a global consensus procedure. There are also problems regarding language barriers in an international consensus meeting. To check for interpretation errors in the Delphi method and to ensure a pragmatic and well-defined set of outcomes the results of the Delphi study will be discussed in an (international) expert meeting. The influence of this meeting on which outcomes are selected for the final COS is however very limited, as this is decided in the prior Delphi study.

**Involving parents and their children**

Involving patients in COS development has recently become common practice with 88% (n=112 as of April 12$^{th}$ 2016) of ongoing COS development studies doing so[18]. Involving patients as participants seems imperative as patients may identify different outcomes that should be measured, compared with physicians[16]. For this protocol we performed a scoping review [unpublished work] that found 12 studies that directly engaged children in COS development. Either as part of the advisory group or the steering committee, or as a stakeholder group in the Delphi[15,22], focus groups[32], interviews[33] or as a part of the consensus meeting[34]. Attempts to engage children and young people in an online Delphi questionnaire have proven to be difficult. In the UK COS for uncomplicated appendicitis,



For peer review only - http://bmjopen.bmj.com/site/about/guidelines.xhtml

there were substantial difficulties with retaining young people in the consecutive rounds of the Delphi questionnaire, despite extensive efforts to optimize the methodology to appeal to children and young people, including: preliminary semi-structured interviews on the subject, pre-testing of the Delphi survey by young people and children[15] and video animations explaining the need for a COS. Parent participation however showed more promising results. Consequently, to safeguard the input of children/young people, the Delphi questionnaire for this study will be developed to be completed by parents with input from their children (5-18 years) whenever possible. In order to ensure that there are no large discrepancies between the opinions of parents with their children, and with children without their parents, we will organize a form of in-person interaction with young people (12-18 years) who have been treated for appendicitis. Involving children/young people in COS development is a subject of interest in many ongoing COS development projects. As the search for the optimal approach to engage young people is ongoing we have not yet selected a final methodology. Two members of the study management group are currently involved in group that is developing such methodology in consultation with young people themselves. We will update our protocol as soon as we settle on a methodology before starting the face-to-face engagement. The updated protocol will be published on an online, open source format (via the Open Science Framework).

A limitation is that due to the international nature of our study it will not be feasible to engage children directly in all the participating countries. That is why the face-to-face engagement will take place in selected countries.

**Other stakeholders**

After careful consideration and consultation with the participating countries, it was decided not to include paediatricians, general practitioners, nurses or emergency medicine physicians. Although all these specialists play an intricate role in the diagnosis and care for children with appendicitis, they do not make the final decision regarding treatment or its provision. We will however, depending on the organisation of the healthcare system in each country, ask these stakeholders to comment on the final COS in order to ensure that essential outcomes are not missed. Since almost all research regarding treatment of paediatric uncomplicated appendicitis is initiated by (paediatric) surgeons, it was decided that researchers will not be included as a separate individual stakeholder group. However, involvement in research will be registered. Whilst their opinion is vital to the development of a COS, it is likely researchers will be well represented in the (paediatric) surgeon stakeholder group. A stratified analyses will be performed to check for skewing of the results by surgeons involved in research. It was also decided not to include journal editors or healthcare commissioners. Even though their opinion is of great importance especially regarding



For peer review only - http://bmjopen.bmj.com/site/about/guidelines.xhtml

implementation, it was determined that their opinion is not essential in establishing the outcomes selected for the COS. There is much variability between countries regarding the role of these stakeholders, which would lead to major challenges regarding Delphi analyses of such a small stakeholder group. However, to enhance implementation and because of their expertise on the use of COSs, representatives from these stakeholder groups will be asked to attend the final consensus discussion.

**Outcome measures**

This study will not answer the question on how to measure the outcomes that are included in the final COS, or at what time point the outcomes should be measured. We will however attempt to come to a clear definition of each outcome. We expect that further research will be necessary to answer the question of timing and how to measure the outcomes. We will advise on this subject in the final report.



For peer review only - http://bmjopen.bmj.com/site/about/guidelines.xhtml

# FOOTNOTES

**Paediatric appendicitis COS development group.**
Minneci PC, MD, Svensson JF, MD, PhD, Luks FI, MD, PhD, St. Peter SD, MD, Abbo O, MD, Arnaud AP, MD, Adams S, MBBS, FRACS, Nah SA, MBBS, MRCS, Skarsgard ED, MD, Pierro A, MD, PhD, Zani A, MD, PhD, Emil S, MD, CM, Keijzer R, MD, PhD, Suominen JS, MD, PhD, Aziz DA, MD, FRCS

**Collaborators:**
The pediatric surgery departments of following hospitals have initiated the COS project and will contribute by recruiting participants. Nationwide Children's Hospital, Columbus, OH, USA. Hasbro Children's Hospital and Alpert Medical School of Brown University, Providence, RI, USA. Children's Mercy Hospital, Kansas City, MO, USA. Karolinska University Hospital, Stockholm, Sweden. Southampton General Hospital, Southampton, UK. Hôpital des Enfants, Centre Hospitalier Universitaire Toulouse, Toulouse, France. Hôpital Femme-Enfant, University Hospital, CHU Rennes, Rennes, France. Sydney Children's Hospital, Randwick NSW, Australia. KK Women's and Children's Hospital, Singapore. BC Children's Hospital, Vancouver, BC, Canada. The Hospital for Sick Children, Toronto, ON, Canada. Montreal Children's Hospital, Montreal, QC, Canada. Emma Children's Hospital, Amsterdam UMC, University of Amsterdam, Vrije Universiteit, Amsterdam, Netherlands. Helsinki Children's Hospital, Helsinki, Finland. Universiti Kebangsaan Malaysia Medical Centre (UKMMC), Kuala Lumpur, Malaysia.

**Acknowledgments**
We acknowledge Hester Rippen as the representative of the Dutch Foundation Children and Hospital for her advice and support in drafting the protocol.

**Author contributions:**
All authors have contributed to the design of this protocol. MK, NJH JHvdL, RB and RRG have initiated the project. The protocol was drafted by MK which was refined by NJH, JHvdL, RB, MO, NJB, LWEvH, and RRG. Statistical advice was provided by JHvdL. MK was responsible for drafting this manuscript. All authors have contributed to the manuscript and read and approved the final manuscript. The paediatric appendicitis COS development group consist of all local investigators who are responsible for translation, ethical board approval, participant recruitment. They have all read, refined and approved the final manuscript.

**Data sharing statement:**
The project is registered on the comet-initiative.org which is open access. The study findings will be presented in a report which will be submitted for publication in a relevant peer-reviewed journal to ensure dissemination to relevant healthcare professionals. Findings may also be submitted for presentation at local meetings or conferences. The protocol will be published on an open access repository.

**Funding statement**: This research received no specific grant from any funding agency in the public, commercial or not-for-profit sectors

**Competing interests statement:** None to declare



For peer review only - http://bmjopen.bmj.com/site/about/guidelines.xhtml

# REFERENCES

1 Addiss DG, Shaffer N, Fowler BS, *et al.* the Epidemiology of Appendicitis and Appendectomy in the United States. *Am J Epidemiol* 1990;**132**:910–25. doi:10.1093/oxfordjournals.aje.a115734

2 Anderson JE, Bickler SW, Chang DC, *et al.* Examining a common disease with unknown etiology: trends in epidemiology and surgical management of appendicitis in California, 1995-2009. *World J Surg* 2012;**36**:2787–94. doi:10.1007/s00268-012-1749-z

3 Ferris M, Quan S, Kaplan BS, *et al.* The Global Incidence of Appendicitis. *Ann Surg* 2017;**266**:237–41. doi:10.1097/SLA.0000000000002188

4 Georgiou R, Eaton S, Stanton MP, *et al.* Efficacy and Safety of Nonoperative Treatment for Acute Appendicitis: A Meta-analysis. *Pediatrics* 2017;:e20163003. doi:10.1542/peds.2016-3003

5 Huang L, Yin Y, Yang L, *et al.* Comparison of Antibiotic Therapy and Appendectomy for Acute Uncomplicated Appendicitis in Children. *JAMA Pediatr* 2017;**171**:426. doi:10.1001/jamapediatrics.2017.0057

6 Xu J, Adams S, Liu YC, *et al.* Nonoperative management in children with early acute appendicitis: A systematic review. *J Pediatr Surg* Published Online First: May 2017. doi:10.1016/j.jpedsurg.2017.05.003

7 Kessler U, Mosbahi S, Walker B, *et al.* Conservative treatment versus surgery for uncomplicated appendicitis in children: a systematic review and meta-analysis. *Arch Dis Child* 2017;:archdischild-2017-313127. doi:10.1136/archdischild-2017-313127

8 Gorter RR, The S-MML, Gorter-Stam MAW, *et al.* Systematic review of nonoperative versus operative treatment of uncomplicated appendicitis. *J Pediatr Surg* 2017;**18 Apr 201**. doi:10.1016/j.jpedsurg.2017.04.005

9 Sinha I, Jones L, Smyth RL, *et al.* A Systematic Review of Studies That Aim to Determine Which Outcomes to Measure in Clinical Trials in Children. *PLoS Med* 2008;**5**:e96. doi:10.1371/journal.pmed.0050096

10 Kirkham JJ, Dwan KM, Altman DG, *et al.* The impact of outcome reporting bias in randomised controlled trials on a cohort of systematic reviews. *BMJ* 2010;**340**:c365.



For peer review only - http://bmjopen.bmj.com/site/about/guidelines.xhtml

11 Pogue J, Yusuf S. Overcoming the limitations of current meta-analysis of randomised controlled trials. *Lancet (London, England)* 1998;**351**:47–52. doi:10.1016/S0140-6736(97)08461-4

12 Zanchetti A, Mancia G. Searching for information from unreported trials--amnesty for the past and prospective meta-analyses for the future. *J Hypertens* 1998;**16**:125.

13 Hall NJ, Kapadia MZ, Eaton S, *et al.* Outcome reporting in randomised controlled trials and meta-analyses of appendicitis treatments in children: a systematic review. *Trials* 2015;**16**:275. doi:10.1186/s13063-015-0783-1

14 Clarke M. Standardising outcomes for clinical trials and systematic reviews. *Trials* 2007;**8**:39. doi:10.1186/1745-6215-8-39

15 Sherratt FC, Eaton S, Walker E, *et al.* Development of a core outcome set to determine the overall treatment success of acute uncomplicated appendicitis in children: a study protocol. *BMJ Paediatr Open* 2017;**1**:e000151. doi:10.1136/bmjpo-2017-000151

16 Sanderson T, Morris M, Calnan M, *et al.* What outcomes from pharmacologic treatments are important to people with rheumatoid arthritis? Creating the basis of a patient core set. *Arthritis Care Res (Hoboken)* 2010;**62**:640–6. doi:10.1002/acr.20034

17 Kirkham JJ, Davis K, Altman DG, *et al.* Core Outcome Set-STAndards for Development: The COS-STAD recommendations. *PLOS Med* 2017;**14**:e1002447. doi:10.1371/journal.pmed.1002447

18 Williamson PR, Altman DG, Bagley H, *et al.* The COMET Handbook: version 1.0. *Trials* 2017;**18**:280. doi:10.1186/s13063-017-1978-4

19 Kirkham JJ, Gorst S, Altman DG, *et al.* Core Outcome Set-STAndards for Reporting: The COS-STAR Statement. *PLoS Med* 2016;**13**:e1002148. doi:10.1371/journal.pmed.1002148

20 Staniszewska S, Brett J, Simera I, *et al.* GRIPP2 reporting checklists: tools to improve reporting of patient and public involvement in research. *BMJ* 2017;**358**:j3453.

21 Protocol for the development of a global core outcome set for treatment of uncomplicated appendicitis in children :: Core Outcome Measures in Effectiveness Trials Initiative (COMET). http://www.comet-initiative.org/studies/details/1119 (accessed 5 Mar 2018).

22 Sinha IP, Smyth RL, Williamson PR. Using the Delphi Technique to Determine Which

For peer review only

Outcomes to Measure in Clinical Trials: Recommendations for the Future Based on a Systematic Review of Existing Studies. *PLoS Med* 2011;**8**:e1000393. doi:10.1371/journal.pmed.1000393

23 Liberati A, Altman DG, Tetzlaff J, *et al.* The PRISMA statement for reporting systematic reviews and meta-analyses of studies that evaluate health care interventions: explanation and elaboration. *J Clin Epidemiol* 2009;**62**:e1–34. doi:10.1016/j.jclinepi.2009.06.006

24 Boers M, Kirwan JR, Gossec L, *et al.* How to choose core outcome measurement sets for clinical trials: OMERACT 11 approves filter 2.0. *J Rheumatol* 2014;**41**:1025–30. doi:10.3899/jrheum.131314

25 Mullen PM. Delphi: myths and reality. *J Health Organ Manag* 2003;**17**:37–52. doi:10.1108/14777260310469319

26 Murphy MK, Black NA, Lamping DL, *et al.* Consensus development methods, and their use in clinical guideline development. *Health Technol Assess* 1998;**2**:i–iv, 1-88.

27 COMET DelphiManager. 2017.http://www.comet-initiative.org/delphimanager/ (accessed 4 Dec 2018).

28 Guyatt GH, Oxman AD, Kunz R, *et al.* GRADE guidelines: 2. Framing the question and deciding on important outcomes. *J Clin Epidemiol* 2011;**64**:395–400. doi:10.1016/j.jclinepi.2010.09.012

29 Brookes ST, Macefield RC, Williamson PR, *et al.* Three nested randomized controlled trials of peer-only or multiple stakeholder group feedback within Delphi surveys during core outcome and information set development. *Trials* 2016;**17**:409. doi:10.1186/s13063-016-1479-x

30 Dodd S, Clarke M, Becker L, *et al.* A taxonomy has been developed for outcomes in medical research to help improve knowledge discovery. *J Clin Epidemiol* 2018;**96**:84–92. doi:10.1016/j.jclinepi.2017.12.020

31 Biggane AM, Brading L, Ravaud P, *et al.* Survey indicated that core outcome set development is increasingly including patients, being conducted internationally and using Delphi surveys. *Trials* 2018;**19**:113. doi:10.1186/s13063-018-2493-y

32 Tsichlaki A, O'Brien K, Johal A, *et al.* Development of a core outcome set for orthodontic trials using a mixed-methods approach: protocol for a multicentre study. *Trials* 2017;**18**:366. doi:10.1186/s13063-017-2098-x



For peer review only - http://bmjopen.bmj.com/site/about/guidelines.xhtml

33 Allard A, Fellowes A, Shilling V, *et al.* Key health outcomes for children and young people with neurodisability: qualitative research with young people and parents. *BMJ Open* 2014;**4**:e004611. doi:10.1136/bmjopen-2013-004611

34 Morris C, Janssens A, Shilling V, *et al.* Meaningful health outcomes for paediatric neurodisability: Stakeholder prioritisation and appropriateness of patient reported outcome measures. *Health Qual Life Outcomes* 2015;**13**:87. doi:10.1186/s12955-015-0284-7

For peer review only



For peer review only - http://bmjopen.bmj.com/site/about/guidelines.xhtml

## Figures

**Figure 1. Schematic depiction of outcome term selection from systematic reviews**
*RCTs= Randomized controlled trials. SRs= Systematic reviews*

For peer review only


For peer review only - http://bmjopen.bmj.com/site/about/guidelines.xhtml

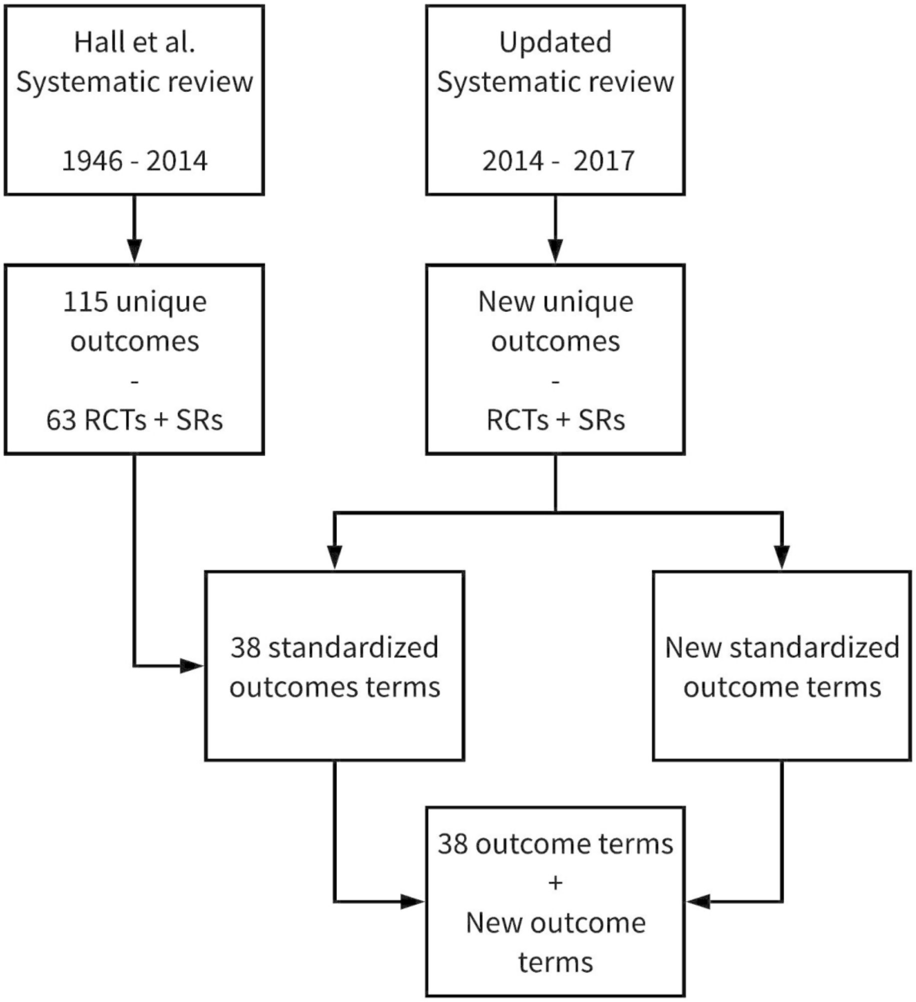


Figure 1. Schematic depiction of outcome term selection from systematic reviews
RCTs= Randomized controlled trials. SRs= Systematic reviews

134x146mm (300 x 300 DPI)

**Online supplement 1.**

S1. Completed COS-STAD checklist for PA-COS project

| Domain | Methodology | Notes | Addressed on page number |
|---|---|---|---|
| Scope specification | 1. The research or practice setting(s) in which the COS is to be applied | e.g., for application in research studies or for use in routine care | 4. |
| | 2. The health condition(s) covered by the COS | e.g., treatment of rheumatoid arthritis or screening for cancer | 4. |
| | 3. The population(s) covered by the COS | e.g., patients with advanced disease or children | 4. |
| | 4. The intervention(s) covered by the COS | e.g., all interventions, drug therapy, or surgical interventions | 4. |
| Stakeholders involved | 5. Those who will use the COS in research | e.g., clinical trialists or industry | 6. |
| | 6. Healthcare professionals with experience of patients with the condition | e.g., clinical experts, practitioners, and investigators with particular experience in the condition | 6. |
| | 7. Patients with the condition or their representatives | involve those who have experienced or who are affected by the condition (e.g., patients, family members, and carers). | 6. |
| Consensus process | 8. The initial list of outcomes considered both healthcare professionals' and patients' views. | consider the views of healthcare professionals and patients (most likely identified from literature reviews or interviews) when generating an initial list of outcomes for inclusion in the consensus process. | 5. (No patient involvement) |
| | 9. A scoring process and consensus definition were described a priori. | Although different consensus methods may be employed in different studies, to avoid any potential biases, COS developers should describe their consensus method a priori. | 7, 8, 9. |
| | 10. Criteria for including/dropping/adding | prespecify criteria for including, dropping, or adding new outcomes to avoid potential biases. | 7, 8, 9. |
| | 11. Care was taken to avoid ambiguity of language used in the list of outcomes. | consider the language used when describing outcomes in front of different stakeholder groups. An example of 1 approach taken is to include both lay and medical terms, with these previously piloted with the stakeholders. | 7,8. |

For peer review only

**Online supplement 2.**

S2. Search strategy for systematic review PA-COS

**Appendicitis**

“Appendix"[Mesh] OR appendix[tiab] OR appendix[ot] OR "Appendicitis"[Mesh] OR "Appendectomy"[Mesh] OR appendicit*[tiab] OR appendicit*[ot] OR appendectom*[tiab] OR appendectom*[ot] OR appendicectom*[tiab] OR appendicectom*[ot]

**Children**

child*[tw] OR schoolchild*[tw] OR infan*[tw] OR adolescen*[tw] OR pediatri*[tw] OR paediatr*[tw] OR neonat*[tw] OR boy[tw] OR boys[tw] OR boyhood[tw] OR girl[tw] OR girls[tw] OR girlhood[tw] OR youth[tw] OR youths[tw] OR baby[tw] OR babies[tw] OR toddler*[tw] OR teen[tw] OR teens[tw] OR teenager*[tw] OR newborn*[tw] OR postneonat*[tw] OR postnat*[tw] OR perinat*[tw] OR puberty[tw] OR preschool*[tw] OR suckling*[tw] OR picu[tw] OR nicu[tw]

**3.2.2 Study selection**

Selection of studies will be performed by 2 independent reviewers (MK, JF) according to the below mentioned in- and exclusion criteria. In case of disagreement between two reviewers, a third independent reviewer (RG) will make the final decision.

*Inclusion/Exclusion Criteria:*

All RCTs and systematic reviews/meta-analyses reporting the outcome of treatment of acute uncomplicated appendicitis will be included in this systematic review. By including systematic reviews that also report on non-comparative studies we expect to identify all reported treatment outcomes, including those from the relatively new field of non-operative management of uncomplicated appendicitis. Publications before January 2014 will be excluded. Only studies in children (<18 years of age) will be included. Studies only reporting on the outcome of treatment in complex or complicated appendicitis (gangrenous appendicitis, appendiceal mass, appendiceal abscess) will be excluded.

**3.2.3 Data extraction**

The two reviewers will extract the data independently using the predefined data extraction form shown in Appendix A. In case of disagreement a third reviewer will make the final decision. A risk of bias assessment of the individual studies is not applicable as we will not be using individual study data but only the reported outcomes. As diversity in terminology will be anticipated, we decided to initially report all outcome measure as mentioned in the original study. Outcome measures will be mapped independently by two reviewers and in case of disagreement a third reviewer will make the final decision. After data extraction of all studies is completed, a meeting of the study management group will be held between to discuss potential similarity between the outcome measures in order to assign an appropriate term for them.

For peer review only

BMJ Open

# Establishing a core outcome set for treatment of uncomplicated appendicitis in children: study protocol for an international Delphi survey.

| | |
|---|---|
| Journal: | *BMJ Open* |
| Manuscript ID | bmjopen-2018-028861.R2 |
| Article Type: | Protocol |
| Date Submitted by the Author: | 17-Apr-2019 |
| Complete List of Authors: | Knaapen, Max; Emma Children’s Hospital, Amsterdam UMC, University of Amsterdam, Vrije Universiteit, Paediatric Surgical Centre<br>Hall, Nigel; University of Southampton Faculty of Medicine, University Surgery Unit<br>van der Lee, Johanna H.; Emma Children’s Hospital, Amsterdam UMC, University of Amsterdam, Paediatric clinical Research Office<br>Butcher, Nancy; Sick Kids, Child Health Evaluative Sciences<br>Offringa, Martin; The Hospital for Sick Children, University of Toronto, Department of Paediatrics<br>Van Heurn, Ernst; Emma Children’s Hospital, Amsterdam UMC, University of Amsterdam, Vrije Universiteit, Paediatric Surgical Centre<br>Bakx, Roel; Emma Children’s Hospital, Amsterdam UMC, University of Amsterdam, Vrije Universiteit, Paediatric Surgical Centre<br>Gorter, Ramon; Emma Children’s Hospital, Amsterdam UMC, University of Amsterdam, Vrije Universiteit, Paediatric Surgical Centre<br>+ On behalf of the paediatric , appendicitis COS development group |
| <b>Primary Subject Heading</b>: | Surgery |
| Secondary Subject Heading: | Emergency medicine, Gastroenterology and hepatology, Medical publishing and peer review, Paediatrics, Research methods |
| Keywords: | delphi, appendicitis, children, core outcome set, paediatric, Global |
| | |

SCHOLARONE™
Manuscripts

# Establishing a core outcome set for treatment of uncomplicated appendicitis in children: study protocol for an international Delphi survey.

Knaapen M, MD[1], Hall NJ, MD, PhD[2], Van der Lee JH, MD, PhD[3], Butcher NJ, PhD[4], Offringa M, MD, PhD[4, 5], Van Heurn, Ernst W.E, PhD[1], Bakx R, MD, PhD[1], Gorter RR, MD, PhD[1] + On behalf of the paediatric appendicitis COS development group

1. Emma Children's Hospital, Amsterdam UMC, University of Amsterdam & Vrije Universiteit, Department of Paediatric Surgery, Amsterdam, The Netherlands. Electronic address: rr.gorter@vumc.nl / r.bakx@amc.uva.nl / m.knaapen@uva.amc.nl / e.vanheurn@amc.uva.nl
2. University Surgery Unit, Faculty of Medicine, University of Southampton, Southampton General Hospital, Southampton, UK; Department of Paediatric Surgery and Urology, Southampton Children's Hospital, Southampton, UK. Electronic address: n.j.hall@soton.ac.uk
3. Emma Children's Hospital, Amsterdam UMC, University of Amsterdam, Paediatric clinical Research Office, Amsterdam, The Netherlands. Electronic address: j.h.vanderlee@amc.uva.nl
4. Child Health Evaluative Sciences, The Hospital for Sick Children Research Institute, Toronto, Canada; Electronic address: nancy.butcher@sickkids.ca
5. Department of Paediatrics, The Hospital for Sick Children, University of Toronto, Toronto, Canada. Electronic address: martin.offringa@sickkids.ca

Address correspondence to Max Knaapen, MD

Address: Emma Children's Hospital, Amsterdam UMC, University of Amsterdam, Vrije Universiteit, Paediatric Surgical Centre, Amsterdam. PO Box 22660, 1100DD Amsterdam, the Netherlands.

Email: M.Knaapen@AMC.UvA.nl

Telephone: +31-20-5665693

Word count: 5885

Funding statement: None

Competing interests statement: None to declare

For peer review only



For peer review only - http://bmjopen.bmj.com/site/about/guidelines.xhtml

# ABSTRACT


## Introduction

Appendicitis is a global disease affecting roughly one in every 12 people in the world, with the highest incidence between ages 10 and 19 years. To date, a wide variety of health outcomes have been reported in randomized controlled trials (RCTs) and meta-analyses evaluating treatments for appendicitis. This is especially the case in studies comparing non-operative treatment to operative treatment. A set of standard outcomes, to be reported in all future trials, is needed to allow for adequate comparison and interpretation of clinical trial results and to make data pooling possible. This protocol describes the development of such a global core outcome set (COS) to allow unified reporting of treatment interventions in children with acute uncomplicated appendicitis.

## Methods and analysis

We use current international standard methodology for the development and reporting of this COS. Its development consists of three phases: (1) Update the most recent systematic review on outcomes reported in uncomplicated paediatric appendicitis research, to identify additional outcomes, (2) Three-step global Delphi study to identify a set of core outcomes for which there is consensus between parents and (paediatric) surgeons, and (3) Expert meeting to finalize the COS and its definitions. Children and young people will be involved through their parents during phase two and will be engaged directly using a customized face-to-face approach.

## Ethics and dissemination.

The medical research ethics committee of the Academic Medical Centre Amsterdam has approved the study. Each participating country/research group will ascertain ethics board approval. Electronic informed consent will be obtained from all participants. Results will be presented in peer-reviewed academic journals and at (international) conferences.

## Registration details

The COS development project was registered with the COMET initiative in February 2018 (http://www.comet-initiative.org/studies/details/1119).


## Strengths and limitations of this study

1. This protocol describes an international online Delhi study that should result in a globally relevant set of core outcomes for paediatric uncomplicated appendicitis.
2.The protocol was developed in conjunction with an international steering committee, patient representation and follows all relevant core outcomes set development guidelines and standards.
3. This study involves parents and patients in deciding what to measure in future uncomplicated appendicitis research.



For peer review only - http://bmjopen.bmj.com/site/about/guidelines.xhtml

4. The involvement of young people in core outcome set development requires a customized approach. This protocol addresses this issue and describes a direct face-to-face involvement.

5. Because of the global and multilingual aspect of the study there will be a limited consensus discussion with only selected individuals. Also, due to feasibility, the direct face-to-face engagement of young people will only take place in selected countries.

For peer review only



For peer review only - http://bmjopen.bmj.com/site/about/guidelines.xhtml

# INTRODUCTION

Appendicitis is a common gastro-intestinal disease affecting roughly one in every 12 people in the world, with the highest incidence between ages 10 and 19 years[1,2]. While the incidence varies from country to country, appendicitis is a global disease[3]. In the last decade, there have been several developments in the treatment of appendicitis in children, with the most recent being non-operative treatment (NOT) for acute uncomplicated appendicitis. Studies investigating the effectiveness of NOT in children show promising results[4–7]. However, the selected primary (and secondary) outcomes vary widely, as reflected in recent systematic reviews assessing the efficacy and safety of NOT, which may contribute to their contradictory conclusions[4–8]. In the systematic review by Georgiou et al.[4], the need for universal outcome selection and reporting in appendicitis studies is emphasized. In general, it is recognized that clinical trials in children often lack outcomes that are appropriately chosen for this particular population[9].

Inconsistent selection and reporting of outcomes limits the ability to adequately compare and interpret clinical trial results. Furthermore, it hampers data pooling and subsequent meta-analysis. It also increases the risk of selective outcome reporting, a form of publication bias. This in turn jeopardizes the validity of results from individual trials, which feeds into subsequent systematic reviews[10] and meta-analyses, which are by nature retrospective, and therefore liable to various risks of bias[11,12].

As demonstrated by Hall et al. in 2015, a wide variety of outcomes has been reported in randomized controlled trials (RCTs) and meta-analyses reporting on the treatment of appendicitis in children[13]. In the 63 included studies, a total of 115 different outcomes were reported[13]. Hall et al. proposed the development of a Core Outcome Set (COS), which is a standardized collection of outcomes that should be measured and reported in all future trials[14]. Recently a study protocol was published for developing such a COS in the United Kingdom[15]. Because of the differences between countries in treatment practises, resources and cultural aspects it was decided, in conjunction with the UK COS research group, that there is a need for an international COS, to be used in all trials assessing the treatment of acute uncomplicated appendicitis in children. The development of the current international protocol was performed in conjunction with the UK research group. Its principal investigator (NJ Hall) has been involved in its development and is part of the study management group.

Outcomes considered important by patients and families are essential to a meaningful and complete COS[16]. That is why parents and patients play a central role in the consensus process as a stakeholder group. Parent and patient representation was ensured through involvement of the Dutch patient and parent Foundation: “Children and Hospital”. A



For peer review only - http://bmjopen.bmj.com/site/about/guidelines.xhtml

representative from this group provided feedback from the perspective of parents and children in several stages of the protocol development. They are also involved in the development of a face-to-face methodology for engaging children in this COS project.

## Scope

We aim to reach a global consensus amongst patients, parents, researchers and physicians on a minimal set of core outcomes that should be measured and reported in all future clinical trials investigating any type of treatment for acute uncomplicated appendicitis in children, including surgical treatment, non-operative treatment or other treatments aimed at curing appendicitis.

# METHODS

In the development of this protocol, we adhere to the COS-STAD (Core Outcome Set-STAndards for Development) recommendations[17] and the COMET (Core Outcome Measures in Effectiveness Trials) handbook[18]. The completed COS-STAD checklist can be found in online supplement S1. The final core outcome set will be reported in accordance with the COS-STAR (COS-STAndaRds for reporting) statement[19]. Involvement of patients and the public will be described using the GRIPP2 reporting checklist[20] (Guidance for Reporting on Involvement of Patients and Public). This study was registered with the COMET initiative (registration number: 1119) on February 11, 2018[21].

### Study design

The paediatric appendicitis COS (PA-COS) development will consist of three phases: (1) An update of the 2015 systematic review on outcomes reported in uncomplicated paediatric appendicitis research[13]. Aiming to identify any additional outcomes used in trials that were published since the previous systematic review; (2) A three-step Delphi study to identify a set of core outcomes from those selected in the literature review. Development of the Delphi is performed according to the checklist by Sinha et al.[22] on the design and reporting of Delphi studies concerning COS selection; and (3) An expert panel meeting including physicians, researchers and children/parent representatives in order to ratify the final COS. Children and young people will be involved through their parents during phase two and will be engaged directly using a customized approach.

### Steering Committee

An international steering committee has been established and consists of the following; the authors, a parent/patient representative of the Dutch Foundation: “Children and Hospital”,

For peer review only



For peer review only - http://bmjopen.bmj.com/site/about/guidelines.xhtml

and the lead local investigator of each participating centre (paediatric appendicitis COS development group). The steering committee will agree on the final version of the protocol at the start of the project and will provide input throughout the duration of the project. The steering committee members will also be involved in the development of the final COS. Within the steering committee, a smaller study management group has been appointed which will convene during regular (videoconference) meetings.

## Systematic review: Treatment outcomes

Hall et al. performed a systematic review of RCTs and meta-analyses reporting treatment outcomes of children with appendicitis up to April 2014[13]. They reported 115 unique outcomes which were collapsed into a total of 38 standardized outcome terms. We will update the systematic review in order to identify any new unique outcomes in clinical trials or systematic reviews. All RCTs and systematic reviews/meta-analyses reporting treatment outcomes of acute uncomplicated appendicitis in children (<18 years of age) published between January 1st 2014 and November 23th 2017 will be included. The final review will follow the PRISMA reporting guideline[23]. We will search the Cochrane Central Register of Controlled Trials, MEDLINE and EMBASE with the help of a clinical librarian. Additional information on the search strategy/study selection and data extraction can be found in online supplement S2. Studies only reporting outcomes of treatment in complex or complicated appendicitis (for example - gangrenous or perforated appendicitis, appendiceal mass, appendiceal abscess) will be excluded.

After data extraction, a meeting of the study management group (including NJ Hall) will be held to discuss potential similarities between the outcomes from the 2015 systematic review from Hall et al.[13]. New unique outcomes will be discussed within the group in order to assign an appropriate standardized outcome term. If these outcomes do not match any of the original 38 outcome terms a new term will be assigned, this methodology is illustrated in figure 1. The new and original outcome terms will be mapped to four core areas (death, life impact, resource use, pathophysiological manifestations) in accordance with the methods from the OMERACT FILTER 2.0[24]. Although Hall et al.[13] chose to list the adverse events as a separate core area, we will reclassify these outcome terms to one of the four core areas (Table 1.). Adverse events of treatment will, however, be labelled separately, as the OMERACT filter suggests[24]. A meeting of the study management group will be held to discuss potential similarities between outcomes and to assign appropriate common outcome terms for corresponding outcomes. Outcomes that are only found once and are not generalizable can be excluded (e.g. the width of lateral thermal damage of the mesoappendix after appendectomy). Grouping the outcomes under a common outcome term aims to arrive at a manageable and cohesive list of outcomes that is appropriate as a basis



For peer review only - http://bmjopen.bmj.com/site/about/guidelines.xhtml

for the Delphi questionnaire.

**Table 1. Outcome Core Areas**

| Core Area | Example(s) |
|---|---|
| Life impact | *Quality of life, loss of ability to work* |
| Resource use | *Length of hospital stay, healthcare costs, societal cost* |
| Pathophysiological Manifestations | *Biochemical parameters, organ function, (ir)reversible manifestations (complications, pathology results)* |
| Death | *Death* |

## Stakeholders and recruitment

### 1) Children and Young People

Children and young people (5-18 years) who have been treated for acute uncomplicated appendicitis in the preceding 24 months, either with initial NOT or with surgery. Children less than 5 years old are excluded as different outcomes might be appropriate in this very young age group. Also, uncomplicated appendicitis is much less common in young children than in older children. Furthermore, there are no studies in which children below the age of 5 are treated non-operatively. Children will be engaged indirectly as we will urge parents to discuss the answers they provide with their child whilst filling out the Delphi questionnaire. Young people will be engaged directly through a customized face-to-face approach in selected countries. For the invited children, considering the complexity of the subject and methodology, age is limited to 12-18 years.

### 2) Parents

Parents of children and young people (5-18 years) treated for acute uncomplicated appendicitis either with initial NOT or with surgery in the preceding 24 months or during the initial phase of the study. Parents will be asked to discuss the answers they provide with their child whilst filling out the Delphi questionnaire. Parents will be



For peer review only - http://bmjopen.bmj.com/site/about/guidelines.xhtml

invited to participate by their child's treating physician or their designate in each participating country/hospital. Participants will be identified retrospectively by contacting patients that were treated in the past 24 months or prospectively by inviting parents to participate after their child has completed its treatment.

3) **(Paediatric) Surgeons**
General and/or paediatric surgeons who care for children in the specified age group will be asked to participate. Surgeons will be identified and invited by the local coordinators in each participating country. These local coordinators are research groups that have previously registered a clinical trial on uncomplicated appendicitis in children. This should allow for inclusion of physicians that also have experience in research on the treatment of appendicitis.

**Participating countries and research groups**

It was decided to invite research groups that are currently conducting clinical trials on the treatment of acute uncomplicated in children. Groups were identified through www.clinicaltrials.gov by searching (January 2017) for 'appendicitis' with an age limitation of 5-18 years. Studies with a mixed population (children and adults) were excluded. Studies that had been completed before 2014, had not been updated since 2015, or with incomplete registrations, were excluded. We found 111 trials, of which 12 trials assessed the treatment of uncomplicated appendicitis in children. Groups from the Netherlands, USA, Canada, Australia, Sweden, Finland, UK, France, Italy, Israel, Japan, Singapore and Malaysia were identified. Some trials included hospitals from multiple countries.

## Sample size

There is no rationale for determining the number of respondents to invite for a Delphi study[18]. A minimum of seven respondents per stakeholder group is suggested to have a large enough group to allow for a consensus process[25]. Taking into account that only some invited participants will register for the Delphi and not all respondents will complete all rounds of the Delphi study (attrition), a minimum of 40 respondents per stakeholder group, per country will be invited. There will be no maximum. In case the number of respondents per country is significantly higher than other countries, we will consider a weightage per country in the analyses. We anticipate that this sample will be large enough to reflect all relevant opinions.

## Delphi study

International online Delphi study


For peer review only - http://bmjopen.bmj.com/site/about/guidelines.xhtml

The Delphi method is an effective tool for reaching consensus in a large group without the need for face-to-face contact[26]. The use of sequential questionnaires which are answered anonymously by stakeholders is an established method for reaching consensus in a group of experts[22]. Questionnaires will be sent using DelphiManager[27], a web-based system designed for Delphi studies. The questionnaires will be open simultaneously to all respondents of the participating countries. After each round, the aggregated responses of all participants are shared anonymously in accordance with the Delphi principle.

The list of outcomes from the systematic review will be formatted into questions, accompanied by an extensive plain language summary per outcome, including figures if appropriate. The Delphi questionnaire will originally be formulated in English and will be translated if required. Translation will only be performed by native speaking professionals.

Participants will be asked to score the importance of each outcome using a 1 to 9 Likert scale as recommended by the Grading of Recommendations Assessment, Development and Evaluation (GRADE) working group[28] and COMET initiative[18]. A score of 7-9 indicates a critical outcome for assessing the effect of a treatment, 4-6 important but not critical, 1-3 indicates an outcome with low importance for assessing the treatment effect. It will also be possible to select an “unable to score” option, which is especially of importance in case parents do not feel equipped to score certain outcomes. The questionnaires, including the plain language summaries, will be piloted by a group of laypersons (n=10) to check for ambiguity and readability.

Delphi round one

Participants will be divided into two stakeholder groups: parents (with their children), and surgeons. Parents will be asked to discuss the answers they provide with their child whilst filling out the Delphi questionnaire. Baseline characteristics (age, country) will be ascertained. Parents will be asked if their child was treated with non-operative or operative treatment, time between registration and the first diagnosis of appendicitis and if their treatment was with or without complications. They will also be asked whether they will be answering the Delphi together with their child. Surgeons will be asked their speciality (paediatric, general, abdominal, other), workplace (academic, teaching hospital, non-teaching hospital), experience with non-operative treatment and experience in research regarding appendicitis in children.

All participants will be asked to score all previously identified outcomes according to their perceived importance for assessing the treatment effect. In the first round there will be an option to suggest additional outcomes not yet listed.

Participants will have between four and eight weeks to complete each round, depending on



For peer review only - http://bmjopen.bmj.com/site/about/guidelines.xhtml

For peer review only

the response rate. In that time they will receive a reminder email every two weeks as long as they have not replied to the questionnaire.

Delphi round one: analysis

Results will be analysed **by stakeholder group and for all participants** using descriptive statistics. Outcomes will be analysed separately for each stakeholder group, as there is evidence that patients are likely to assign importance to outcomes differently than surgeons[29], which has the potential to influence eventual outcome selection.

"Consensus-in" will be defined as:

- Greater than 70% of participants in **both** stakeholder groups scoring the outcome as 7-9 and less than 15% in **both** stakeholder groups scoring the outcome as 1-3.
- Greater than 90% of participants within **one** stakeholder group scoring the outcome as 7-9. This implies that these outcomes are highly regarded by an individual stakeholder group, and should also be included[18].

"Consensus-out" will be defined as:

- Greater than 70% of participants in **both** stakeholder groups scoring the outcomes as 1-3 and less than 15% of participants in **both** stakeholder groups scoring the outcome as 7–9. Consensus-out can only be reached when there is consensus across **both** stakeholder groups.

Outcomes that do not meet any of these criteria will be defined as "no consensus". A stratified analysis will be performed to check for skewing as a result of divergent opinions from a single country, or surgeons with or without research experience.

At the end of round one, there will be a meeting of the study management group to assess whether an alteration in the Delphi study is appropriate. If additional outcomes are suggested by Delphi participants, each outcome will be assessed by the study management group to determine whether it is indeed new and to which category it should be classified. Wording of the Delphi questionnaire will be adjusted if misinterpretation is suspected.

Delphi rounds two and three

All participants that complete the previous round will be asked to participate in the next round. Only outcomes that have not yet been defined as "consensus-in" or "consensus-out" during the previous round will be presented in the following rounds to **all** participants. Outcomes for which there was only "consensus-in" within a single stakeholder group, will still be presented to the other stakeholder group to evaluate whether consensus can be achieved



For peer review only

in both stakeholder groups. An overview of included and excluded outcomes will be available. The outcomes for which there is no consensus and the newly suggested outcomes from the previous rounds will be presented with the participants' individual score and the median scores from each stakeholder group combined with a histogram showing the scoring distribution. Participants will be asked to score all remaining outcomes in the same manner as in round one.

Delphi rounds two and three analysis

Results will be analysed per stakeholder group and for all participants, using descriptive statistics, including a stratified analysis. The same definitions for consensus in/out as in the first Delphi round are upheld. After the second round, there will be a meeting of the study management group to assess the need for alterations in the Delphi study, and to decide whether or not to proceed with a third Delphi round, assuming consensus between **both** stakeholder groups on more than 80% of the outcomes, and more than five outcomes with consensus in. To give an estimate of the degree of agreement between respondents, the width of the interquartile range of the median ranking score will be calculated, potentially ranging from 0.00, meaning complete agreement, to 8.00, meaning least possible agreement. This will be calculated for both the individual stakeholder groups as well as the entire group of respondents after the final round.

## Face-to-face engagement of young people

We wish to check for discrepancies of opinion between parents answering the Delphi together with their child and children who are interviewed directly. For this, a form of in-person interaction will be organised with young people (12-18 years) who have been treated for appendicitis. They will be asked to comment on the preliminary COS selection established at the end of the Delphi study, and to suggest additional outcomes and comment on outcomes that did not make the preliminary COS selection. This will either be done by a short, face-to-face, one round questionnaire involving only outcomes relevant to children/young people, or in the form of a small consensus meeting (prioritization meeting) before finalizing the definitive COS. Doing this type of research requires experienced interviewers and resources. That is why the face-to-face engagement will only take place in selected countries, however, we will aim to involve as many countries as feasible. Separate ethical board approval will be obtained as appropriate.

## Consensus discussion

If adequate consensus (we aim to achieve consensus on at least one outcome per OMERACT core area) is reached in the Delphi study, we will organise a face-to-face expert panel meeting with selected individuals with the purpose to ratify a pragmatic and well-



For peer review only - http://bmjopen.bmj.com/site/about/guidelines.xhtml

defined set of outcomes. A secondary aim of this meeting is to enhance support and implementation of the final COS.

The meeting will be held at an international conference for paediatric surgery. Through purposive sampling, approximately 30 “experts” from across all stakeholder groups, including physicians, researchers and children/parent representatives, will be invited to participate in a face-to-face meeting with the Steering Committee. Journal editors and healthcare commissioners will also be invited to attend in an observational capacity with the purpose of promoting implementation and to provide comments on the final list of outcomes.

In the event that adequate consensus cannot be reached in the Delphi process, we will organise a formal face-to-face consensus meeting or teleconference. In that case, we will select an appropriate representation of all stakeholder groups from the panel members that participated in the Delphi study.

## Final COS development

The goal is to achieve a pragmatic COS that is applicable and feasible for all future trials that evaluate the treatment of uncomplicated appendicitis in children. There is no recommended maximum number of outcomes that should be included in a COS. However, if the final COS includes to many outcomes, the COS would not be feasible to use in practice. To achieve the goal of a pragmatic COS we aim to arrive at a maximum of 10 outcomes, the same maximum number as the UK COS protocol specifies[15]. As a minimum, we aim to have at least one outcome per core area. If the number of outcomes for which consensus is achieved greatly exceeds 10 outcomes, the outcomes with the highest level of consensus will be considered part of the suggested COS. However, we will report all outcomes for which consensus is achieved. The highest level of consensus depends on whether there is consensus in both stakeholder groups, the median score that was appointed to the outcome, and the interquartile range of the median score as an estimate of the degree of consensus.

Only outcomes for which consensus is reached internationally will be selected. To test for country bias, stratified analyses of the Delphi results will be performed. The results from the face-to-face engagement of young people will be taken into account for the final COS selection and will be reported separately. If there is no consensus between patients, parents and healthcare professionals, an outcome can still be selected if there is clear consensus within a single stakeholder group. These will be reported separately. The final COS will be categorised according to the four core areas of the OMERACT filter[24]. We will also annotate the outcomes according to the recently published outcome taxonomy to maximise future data harmonisation[30].



For peer review only - http://bmjopen.bmj.com/site/about/guidelines.xhtml

## Patient and Public Involvement

Patient involvement is at the core of this study design. By asking parents and patients with experience in having uncomplicated appendicitis what outcomes they feel should be part of future research. To ensure our design is appropriate for parents and children we have involved the Dutch child and parents representation group as part of the steering committee. In that capacities they provide input on the protocol and the study. To make sure the Delphi questionnaire is understandable and has no ambiguities it is checked by a group of laypersons before the start of the Delphi study. Part of the Delphi study is giving feedback to all its participants after each round, this will also be done with the final study results.

## Ethics and dissemination.

Ethics

The medical research ethics committee of the Academic Medical Centre Amsterdam confirmed that the Dutch Medical Research Involving Human Subjects Act (WMO) does not apply to this study and that complete approval of this study by the committee is not required. Each participating country/research group will be asked to obtain ethics board approval or confirm that ethics board approval it not required. Electronic informed consent will be obtained from all participants. The face-to-face engagement of young people (12-18 years) will take place in selected countries and separate ethics board approval will be obtained, as appropriate.

Data collection and confidentiality

All data will be handled confidentially and in accordance with the Dutch Personal Data Protection Act and the European General Data Protection Regulation (GDPR). DelphiManager[27] will be used for the online questionnaire. After informed consent from all participants only limited identifying information (name, email) will be ascertained during registration. This information will be stored separately from the answers given in the questionnaire and will only be used for the purpose of direct feedback and reminder emails. Access to personally identifiable data will be strictly limited.

Study status and dissemination

In the first quarter (Q1) of 2018 the following 13 countries were invited to participate in the project; Netherlands, USA, Canada, Australia, Sweden, Finland, UK, France, Italy, Israel, Japan, Singapore and Malaysia. Ten countries replied, Italy, Israel and Japan did not. In Q1 2018 the systematic review was finished. In Q2 2018 the Delphi questionnaire was developed and piloted. In Q3 2018 all materials were translated. Between Q4 2018 and Q1 2019 IRB applications were submitted in 10 countries and 15 participating centres. The anticipated start of the online Delphi study is May 2019. We anticipate to have the final COS



For peer review only - http://bmjopen.bmj.com/site/about/guidelines.xhtml

ready by Q1 2020. Dissemination of the results will be accomplished by publication in an international peer-reviewed scientific journal and by presentations at (international) conferences. By involving the majority of the principal investigators who are currently involved in research on uncomplicated appendicitis in children, we aim to optimize uptake of the final COS. By involving journal editors and healthcare commissioners in the face-to-face consensus discussion, we aim to ultimately have the COS introduced as a requirement in future outcome reporting on the treatment of uncomplicated appendicitis in children. We will also actively send out the final COS to relevant journal editors and funding bodies to promote uptake in future research.

# DISCUSSION

## Strengths and limitations of this study

### Outcomes selection

The selection of potential outcomes will be done systematically and will provide a selection for the first Delphi questionnaire that reflects most issues pertinent to the treatment of uncomplicated appendicitis. By including systematic reviews/meta-analyses that also report on non-comparative studies, we expect to identify all reported treatment outcomes, including those from the relatively new field of NOT for uncomplicated appendicitis.

To be able to arrive at a manageable list of outcomes that is appropriate for a Delphi study, the number of outcome terms needs to be somewhat limited. In order to achieve this, the outcomes derived from our systematic review will be merged in case of similarity. If outcomes are not generalizable and only reported once, they will be excluded. This will be proposed and prepared by two independent reviewers and discussed in the study management group. However, the merging of outcomes will inevitably lead to some loss of detail.

### Global consensus

In order to reflect the views of different stakeholders, a variety of groups will be part of the development of this COS. This is not only the case on a national level, but also on an international level, related to, for example, differences between countries in resources, treatment practises for acute uncomplicated appendicitis, and cultural differences. For example there is a large difference with regard to the standard length of hospital stay after an appendectomy for uncomplicated appendicitis. In the USA much effort is devoted to reduce the number of admission days, in the UK there is only limited attention for the duration of admission and for instance in Japan an admission for 5 days is not uncommon. We can expect that these kind of differences result in different opinions regarding the core outcomes set. By also involving patients and parents from the participating countries we hope to correct



For peer review only - http://bmjopen.bmj.com/site/about/guidelines.xhtml

for these differences[31]. In conjunction with the UK paediatric appendicitis COS research group we decided that an international validation of the UK COS would not give the depth of information and would not allow for consensus formation on all possible outcomes. Which we feel is appropriate considering the before mentioned significant differences between countries. Involving members from different countries will not only lead to the development of a COS that reflects the opinions of the international community, it should also lead to an internationally applicable “minimal” COS. However, selecting the participating countries on the basis of their involvement in research on appendicitis in children is a limitation. This choice was made on the basis of feasibility. Researchers in the field of uncomplicated appendicitis have an interest in the development of a COS and have the network to help carry out the Delphi study. With our current selection we will still have participants from four different continents. Our method of country selection has another advantage. Since non-operative treatment is an important research subject in childhood appendicitis, we aim to include surgeons and parents who have experience in that field. As non-operative treatment is still experimental in most of the world, we also need surgeons and patients who have been involved in such research.

**Limited face-to-face consensus**

If consensus is reached in the Delphi study we will not be organising a formal consensus meeting. The Delphi method can be used for reaching consensus in a group of respondents without the need for face-to-face contact. There is a risk of bias if a face-to-face consensus meeting leads to selection of only participants who are able to attend the meeting, which is especially a problem in a global consensus procedure. There are also problems regarding language barriers in an international consensus meeting. To check for interpretation errors in the Delphi method and to ensure a pragmatic and well-defined set of outcomes the results of the Delphi study will be discussed in an (international) expert meeting. The influence of this meeting on which outcomes are selected for the final COS is however very limited, as this selection is primarily made in the Delphi study.

**Involving parents and their children**

Involving patients in COS development has recently become common practice with 88% (n=112 as of April 12th 2016) of ongoing COS development studies doing so[18]. Involving patients as participants seems imperative as patients may select different outcomes, compared to physicians[16]. For this protocol we performed a scoping review [unpublished work] that found 12 studies that directly engaged children in COS development. Either as part of the advisory group or the steering committee, or as a stakeholder group in the Delphi[15,22], focus groups[32], interviews[33] or as a part of the consensus meeting[34]. Attempts to engage children and young people in an online Delphi questionnaire have



For peer review only - http://bmjopen.bmj.com/site/about/guidelines.xhtml

proven to be difficult. In the UK COS for uncomplicated appendicitis, there were substantial difficulties with retaining young people in the consecutive rounds of the Delphi questionnaire, despite extensive efforts to optimize the methodology to appeal to children and young people, including: preliminary semi-structured interviews on the subject, pre-testing of the Delphi survey by young people and children[15] and video animations explaining the need for a COS. Parent participation however showed more promising results. Consequently, to safeguard the input of children/young people, the Delphi questionnaire for this study will be developed to be completed by parents with input from their children (5-18 years) whenever possible. In order to ensure that there are no large discrepancies between the opinions of parents together with their child, and with children without their parents, we will organize a form of in-person interaction with young people (12-18 years) who have been treated for appendicitis. Involving children/young people in COS development is a subject of interest in many ongoing COS development projects. As the search for the optimal approach to engage young people is ongoing we have not yet selected a final methodology. Two members of the study management group are currently involved in group that is developing such methodology in consultation with young people themselves. We will update our protocol as soon as we settle on a methodology before starting the face-to-face engagement. The updated protocol will be published on an online, open source format (via the Open Science Framework).

A limitation is that due to the international nature of our study it will not be feasible to engage children directly in all the participating countries. That is why the face-to-face engagement will take place in selected countries.

**Other stakeholders**

After careful consideration and consultation with the participating countries, it was decided not to include paediatricians, general practitioners, nurses or emergency medicine physicians. Although all these specialists play an intricate role in the diagnosis and care for children with appendicitis, they do not make the final decision regarding treatment or its provision. We will however, depending on the organisation of the healthcare system in each country, ask these stakeholders to comment on the final COS in order to ensure that essential outcomes are not missed. Since almost all research regarding treatment of paediatric uncomplicated appendicitis is initiated by (paediatric) surgeons, it was decided that researchers will not be included as a separate individual stakeholder group. However, involvement in research will be registered. Whilst their opinion is vital to the development of a COS, it is likely researchers will be well represented in the (paediatric) surgeon stakeholder group. A stratified analyses will be performed to check for skewing of the results by surgeons involved in research. It was also decided not to include journal editors or healthcare



For peer review only - http://bmjopen.bmj.com/site/about/guidelines.xhtml

commissioners. Even though their opinion is of great importance especially regarding implementation, it was determined that their opinion is not essential in establishing the outcomes selected for the COS. Also there is much variability between countries regarding the role of these stakeholders, which would lead to major challenges regarding Delphi analyses of such a small stakeholder group. However, to enhance implementation and because of their expertise on the use of COSs, representatives of these stakeholder groups will be asked to attend the final consensus discussion.

**Outcome measures**

This study will not answer the question on how to measure the outcomes that are included in the final COS, or at what time point the outcomes should be measured. We will however attempt to come to a clear definition of each outcome. We expect that further research will be necessary to answer the question of timing and how to measure the outcomes. We will advise on this subject in the final report.

For peer review only



For peer review only - http://bmjopen.bmj.com/site/about/guidelines.xhtml

# FOOTNOTES

**Paediatric appendicitis COS development group.**
Minneci PC, MD, Svensson JF, MD, PhD, Luks FI, MD, PhD, St. Peter SD, MD, Abbo O, MD, Arnaud AP, MD, Adams S, MBBS, FRACS, Nah SA, MBBS, MRCS, Skarsgard ED, MD, Pierro A, MD, PhD, Zani A, MD, PhD, Emil S, MD, CM, Keijzer R, MD, PhD, Suominen JS, MD, PhD, Aziz DA, MD, FRCS

**Collaborators:**
The pediatric surgery departments of following hospitals have initiated the COS project and will contribute by recruiting participants. Nationwide Children's Hospital, Columbus, OH, USA. Hasbro Children's Hospital and Alpert Medical School of Brown University, Providence, RI, USA. Children's Mercy Hospital, Kansas City, MO, USA. Karolinska University Hospital, Stockholm, Sweden. Southampton General Hospital, Southampton, UK. Hôpital des Enfants, Centre Hospitalier Universitaire Toulouse, Toulouse, France. Hôpital Femme-Enfant, University Hospital, CHU Rennes, Rennes, France. Sydney Children's Hospital, Randwick NSW, Australia. KK Women's and Children's Hospital, Singapore. BC Children's Hospital, Vancouver, BC, Canada. The Hospital for Sick Children, Toronto, ON, Canada. Montreal Children's Hospital, Montreal, QC, Canada. Emma Children's Hospital, Amsterdam UMC, University of Amsterdam, Vrije Universiteit, Amsterdam, Netherlands. Helsinki Children's Hospital, Helsinki, Finland. Universiti Kebangsaan Malaysia Medical Centre (UKMMC), Kuala Lumpur, Malaysia.

**Study management group**
Knaapen M, Hall NJ, Van der Lee JH, Butcher NJ, Offringa M, Bakx R, Gorter RR

**Acknowledgments**
We acknowledge Hester Rippen as the representative of the Dutch Foundation Children and Hospital for her advice and support in drafting the protocol.

**Author contributions:**
All authors have contributed to the design of this protocol. MK, NJH JHvdL, RB and RRG have initiated the project. The protocol was drafted by MK which was refined by NJH, JHvdL, RB, MO, NJB, EWEvH, and RRG. Statistical advice was provided by JHvdL. MK was responsible for drafting this manuscript. All authors have contributed to the manuscript and read and approved the final manuscript. The paediatric appendicitis COS development group consist of all local investigators who are responsible for translation, ethical board approval, participant recruitment. They have all read, refined and approved the final manuscript.

**Data sharing statement:**
The project is registered on the comet-initiative.org which is open access. The study findings will be presented in a report which will be submitted for publication in a relevant peer-reviewed journal to ensure dissemination to relevant healthcare professionals. Findings may also be submitted for presentation at local meetings or conferences. The protocol will be published on an open access repository.

**Funding statement**: This research received no specific grant from any funding agency in the public, commercial or not-for-profit sectors

**Competing interests statement:** None to declare

For peer review only

# REFERENCES


1 Addiss DG, Shaffer N, Fowler BS, *et al.* the Epidemiology of Appendicitis and Appendectomy in the United States. *Am J Epidemiol* 1990;**132**:910–25. doi:10.1093/oxfordjournals.aje.a115734

2 Anderson JE, Bickler SW, Chang DC, *et al.* Examining a common disease with unknown etiology: trends in epidemiology and surgical management of appendicitis in California, 1995-2009. *World J Surg* 2012;**36**:2787–94. doi:10.1007/s00268-012-1749-z

3 Ferris M, Quan S, Kaplan BS, *et al.* The Global Incidence of Appendicitis. *Ann Surg* 2017;**266**:237–41. doi:10.1097/SLA.0000000000002188

4 Georgiou R, Eaton S, Stanton MP, *et al.* Efficacy and Safety of Nonoperative Treatment for Acute Appendicitis: A Meta-analysis. *Pediatrics* 2017;:e20163003. doi:10.1542/peds.2016-3003

5 Huang L, Yin Y, Yang L, *et al.* Comparison of Antibiotic Therapy and Appendectomy for Acute Uncomplicated Appendicitis in Children. *JAMA Pediatr* 2017;**171**:426. doi:10.1001/jamapediatrics.2017.0057

6 Xu J, Adams S, Liu YC, *et al.* Nonoperative management in children with early acute appendicitis: A systematic review. *J Pediatr Surg* Published Online First: May 2017. doi:10.1016/j.jpedsurg.2017.05.003

7 Kessler U, Mosbahi S, Walker B, *et al.* Conservative treatment versus surgery for uncomplicated appendicitis in children: a systematic review and meta-analysis. *Arch Dis Child* 2017;:archdischild-2017-313127. doi:10.1136/archdischild-2017-313127

8 Gorter RR, The S-MML, Gorter-Stam MAW, *et al.* Systematic review of nonoperative versus operative treatment of uncomplicated appendicitis. *J Pediatr Surg* 2017;**18 Apr 201**. doi:10.1016/j.jpedsurg.2017.04.005

9 Sinha I, Jones L, Smyth RL, *et al.* A Systematic Review of Studies That Aim to Determine Which Outcomes to Measure in Clinical Trials in Children. *PLoS Med* 2008;**5**:e96. doi:10.1371/journal.pmed.0050096

10 Kirkham JJ, Dwan KM, Altman DG, *et al.* The impact of outcome reporting bias in randomised controlled trials on a cohort of systematic reviews. *BMJ* 2010;**340**:c365.




For peer review only - http://bmjopen.bmj.com/site/about/guidelines.xhtml

11 Pogue J, Yusuf S. Overcoming the limitations of current meta-analysis of randomised controlled trials. *Lancet (London, England)* 1998;**351**:47–52. doi:10.1016/S0140-6736(97)08461-4

12 Zanchetti A, Mancia G. Searching for information from unreported trials--amnesty for the past and prospective meta-analyses for the future. *J Hypertens* 1998;**16**:125.

13 Hall NJ, Kapadia MZ, Eaton S, *et al.* Outcome reporting in randomised controlled trials and meta-analyses of appendicitis treatments in children: a systematic review. *Trials* 2015;**16**:275. doi:10.1186/s13063-015-0783-1

14 Clarke M. Standardising outcomes for clinical trials and systematic reviews. *Trials* 2007;**8**:39. doi:10.1186/1745-6215-8-39

15 Sherratt FC, Eaton S, Walker E, *et al.* Development of a core outcome set to determine the overall treatment success of acute uncomplicated appendicitis in children: a study protocol. *BMJ Paediatr Open* 2017;**1**:e000151. doi:10.1136/bmjpo-2017-000151

16 Sanderson T, Morris M, Calnan M, *et al.* What outcomes from pharmacologic treatments are important to people with rheumatoid arthritis? Creating the basis of a patient core set. *Arthritis Care Res (Hoboken)* 2010;**62**:640–6. doi:10.1002/acr.20034

17 Kirkham JJ, Davis K, Altman DG, *et al.* Core Outcome Set-STAndards for Development: The COS-STAD recommendations. *PLOS Med* 2017;**14**:e1002447. doi:10.1371/journal.pmed.1002447

18 Williamson PR, Altman DG, Bagley H, *et al.* The COMET Handbook: version 1.0. *Trials* 2017;**18**:280. doi:10.1186/s13063-017-1978-4

19 Kirkham JJ, Gorst S, Altman DG, *et al.* Core Outcome Set-STAndards for Reporting: The COS-STAR Statement. *PLoS Med* 2016;**13**:e1002148. doi:10.1371/journal.pmed.1002148

20 Staniszewska S, Brett J, Simera I, *et al.* GRIPP2 reporting checklists: tools to improve reporting of patient and public involvement in research. *BMJ* 2017;**358**:j3453.

21 Protocol for the development of a global core outcome set for treatment of uncomplicated appendicitis in children :: Core Outcome Measures in Effectiveness Trials Initiative (COMET). http://www.comet-initiative.org/studies/details/1119 (accessed 5 Mar 2018).

22 Sinha IP, Smyth RL, Williamson PR. Using the Delphi Technique to Determine Which



For peer review only

For peer review only - http://bmjopen.bmj.com/site/about/guidelines.xhtml

Outcomes to Measure in Clinical Trials: Recommendations for the Future Based on a Systematic Review of Existing Studies. *PLoS Med* 2011;**8**:e1000393. doi:10.1371/journal.pmed.1000393

23 Liberati A, Altman DG, Tetzlaff J, *et al.* The PRISMA statement for reporting systematic reviews and meta-analyses of studies that evaluate health care interventions: explanation and elaboration. *J Clin Epidemiol* 2009;**62**:e1–34. doi:10.1016/j.jclinepi.2009.06.006

24 Boers M, Kirwan JR, Gossec L, *et al.* How to choose core outcome measurement sets for clinical trials: OMERACT 11 approves filter 2.0. *J Rheumatol* 2014;**41**:1025–30. doi:10.3899/jrheum.131314

25 Mullen PM. Delphi: myths and reality. *J Health Organ Manag* 2003;**17**:37–52. doi:10.1108/14777260310469319

26 Murphy MK, Black NA, Lamping DL, *et al.* Consensus development methods, and their use in clinical guideline development. *Health Technol Assess* 1998;**2**:i–iv, 1-88.

27 COMET DelphiManager. 2017.http://www.comet-initiative.org/delphimanager/ (accessed 4 Dec 2018).

28 Guyatt GH, Oxman AD, Kunz R, *et al.* GRADE guidelines: 2. Framing the question and deciding on important outcomes. *J Clin Epidemiol* 2011;**64**:395–400. doi:10.1016/j.jclinepi.2010.09.012

29 Brookes ST, Macefield RC, Williamson PR, *et al.* Three nested randomized controlled trials of peer-only or multiple stakeholder group feedback within Delphi surveys during core outcome and information set development. *Trials* 2016;**17**:409. doi:10.1186/s13063-016-1479-x

30 Dodd S, Clarke M, Becker L, *et al.* A taxonomy has been developed for outcomes in medical research to help improve knowledge discovery. *J Clin Epidemiol* 2018;**96**:84–92. doi:10.1016/j.jclinepi.2017.12.020

31 Biggane AM, Brading L, Ravaud P, *et al.* Survey indicated that core outcome set development is increasingly including patients, being conducted internationally and using Delphi surveys. *Trials* 2018;**19**:113. doi:10.1186/s13063-018-2493-y

32 Tsichlaki A, O'Brien K, Johal A, *et al.* Development of a core outcome set for orthodontic trials using a mixed-methods approach: protocol for a multicentre study. *Trials* 2017;**18**:366. doi:10.1186/s13063-017-2098-x

For peer review only



For peer review only - http://bmjopen.bmj.com/site/about/guidelines.xhtml

33 Allard A, Fellowes A, Shilling V, *et al.* Key health outcomes for children and young people with neurodisability: qualitative research with young people and parents. *BMJ Open* 2014;**4**:e004611. doi:10.1136/bmjopen-2013-004611

34 Morris C, Janssens A, Shilling V, *et al.* Meaningful health outcomes for paediatric neurodisability: Stakeholder prioritisation and appropriateness of patient reported outcome measures. *Health Qual Life Outcomes* 2015;**13**:87. doi:10.1186/s12955-015-0284-7

For peer review only

684 # Figures

685

686 **Figure 1. Schematic depiction of outcome term selection from systematic reviews**

687 *RCTs= Randomized controlled trials. SRs= Systematic reviews*

For peer review only



For peer review only - http://bmjopen.bmj.com/site/about/guidelines.xhtml

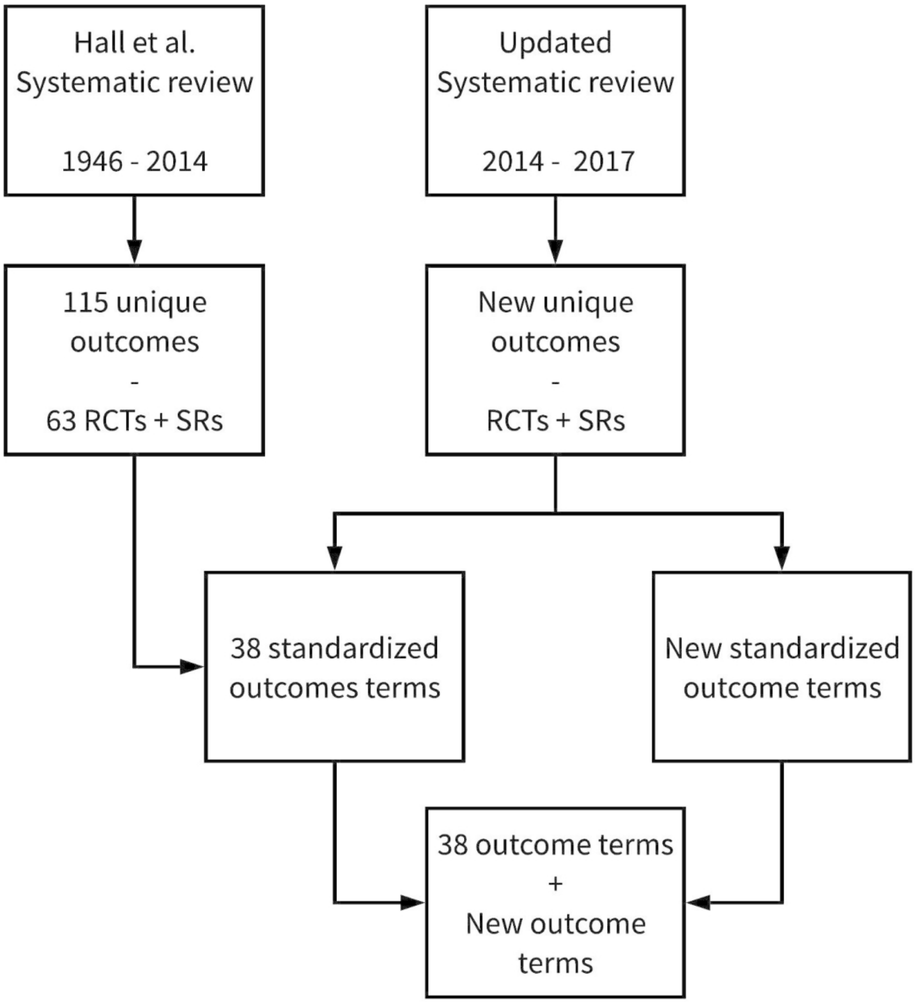


Figure 1. Schematic depiction of outcome term selection from systematic reviews
RCTs= Randomized controlled trials. SRs= Systematic reviews

134x146mm (300 x 300 DPI)

**Online supplement 1.**

S1. Completed COS-STAD checklist for PA-COS project

| Domain | Methodology | Notes | Addressed on page number |
|---|---|---|---|
| Scope specification | 1. The research or practice setting(s) in which the COS is to be applied | e.g., for application in research studies or for use in routine care | 4. |
| | 2. The health condition(s) covered by the COS | e.g., treatment of rheumatoid arthritis or screening for cancer | 4. |
| | 3. The population(s) covered by the COS | e.g., patients with advanced disease or children | 4. |
| | 4. The intervention(s) covered by the COS | e.g., all interventions, drug therapy, or surgical interventions | 4. |
| Stakeholders involved | 5. Those who will use the COS in research | e.g., clinical trialists or industry | 6. |
| | 6. Healthcare professionals with experience of patients with the condition | e.g., clinical experts, practitioners, and investigators with particular experience in the condition | 6. |
| | 7. Patients with the condition or their representatives | involve those who have experienced or who are affected by the condition (e.g., patients, family members, and carers). | 6. |
| Consensus process | 8. The initial list of outcomes considered both healthcare professionals' and patients' views. | consider the views of healthcare professionals and patients (most likely identified from literature reviews or interviews) when generating an initial list of outcomes for inclusion in the consensus process. | 5. (No patient involvement) |
| | 9. A scoring process and consensus definition were described a priori. | Although different consensus methods may be employed in different studies, to avoid any potential biases, COS developers should describe their consensus method a priori. | 7, 8, 9. |
| | 10. Criteria for including/dropping/adding | prespecify criteria for including, dropping, or adding new outcomes to avoid potential biases. | 7, 8, 9. |
| | 11. Care was taken to avoid ambiguity of language used in the list of outcomes. | consider the language used when describing outcomes in front of different stakeholder groups. An example of 1 approach taken is to include both lay and medical terms, with these previously piloted with the stakeholders. | 7,8. |

For peer review only

**Online supplement 2.**

S2. Search strategy for systematic review PA-COS

**Appendicitis**

“Appendix"[Mesh] OR appendix[tiab] OR appendix[ot] OR "Appendicitis"[Mesh] OR "Appendectomy"[Mesh] OR appendicit*[tiab] OR appendicit*[ot] OR appendectom*[tiab] OR appendectom*[ot] OR appendicectom*[tiab] OR appendicectom*[ot]

**Children**

child*[tw] OR schoolchild*[tw] OR infan*[tw] OR adolescen*[tw] OR pediatri*[tw] OR paediatr*[tw] OR neonat*[tw] OR boy[tw] OR boys[tw] OR boyhood[tw] OR girl[tw] OR girls[tw] OR girlhood[tw] OR youth[tw] OR youths[tw] OR baby[tw] OR babies[tw] OR toddler*[tw] OR teen[tw] OR teens[tw] OR teenager*[tw] OR newborn*[tw] OR postneonat*[tw] OR postnat*[tw] OR perinat*[tw] OR puberty[tw] OR preschool*[tw] OR suckling*[tw] OR picu[tw] OR nicu[tw]

**3.2.2 Study selection**

Selection of studies will be performed by 2 independent reviewers (MK, JF) according to the below mentioned in- and exclusion criteria. In case of disagreement between two reviewers, a third independent reviewer (RG) will make the final decision.

*Inclusion/Exclusion Criteria:*

All RCTs and systematic reviews/meta-analyses reporting the outcome of treatment of acute uncomplicated appendicitis will be included in this systematic review. By including systematic reviews that also report on non-comparative studies we expect to identify all reported treatment outcomes, including those from the relatively new field of non-operative management of uncomplicated appendicitis. Publications before January 2014 will be excluded. Only studies in children (<18 years of age) will be included. Studies only reporting on the outcome of treatment in complex or complicated appendicitis (gangrenous appendicitis, appendiceal mass, appendiceal abscess) will be excluded.

**3.2.3 Data extraction**

The two reviewers will extract the data independently using the predefined data extraction form shown in Appendix A. In case of disagreement a third reviewer will make the final decision. A risk of bias assessment of the individual studies is not applicable as we will not be using individual study data but only the reported outcomes. As diversity in terminology will be anticipated, we decided to initially report all outcome measure as mentioned in the original study. Outcome measures will be mapped independently by two reviewers and in case of disagreement a third reviewer will make the final decision. After data extraction of all studies is completed, a meeting of the study management group will be held between to discuss potential similarity between the outcome measures in order to assign an appropriate term for them.



For peer review only - http://bmjopen.bmj.com/site/about/guidelines.xhtml
